# Electrostatic Interactions Are the Primary Determinant of the Binding Affinity of SARS-CoV-2 Spike RBD to ACE2: A Computational Case Study of Omicron Variants

**DOI:** 10.3390/ijms232314796

**Published:** 2022-11-26

**Authors:** Peng Sang, Yong-Qin Chen, Meng-Ting Liu, Yu-Ting Wang, Ting Yue, Yi Li, Yi-Rui Yin, Li-Quan Yang

**Affiliations:** 1 College of Agriculture and Biological Science, Dali University, Dali 671000, China; 2 Key Laboratory of Bioinformatics and Computational Biology, Department of Education of Yunnan Province, Dali University, Dali 671000, China; 3 Yunnan Key Laboratory of Screening and Research on Anti-Pathogenic Plant Resources from West Yunnan, Dali University, Dali 671000, China; 4 College of Mathematics and Computer Science, Dali University, Dali 671000, China

**Keywords:** SARS-CoV-2, Omicron, RBD, ACE2, binding affinity, electrostatic interactions, molecular dynamics simulation, MM-PBSA

## Abstract

To explore the mechanistic origin that determines the binding affinity of SARS-CoV-2 spike receptor binding domain (RBD) to human angiotensin converting enzyme 2 (ACE2), we constructed the homology models of RBD-ACE2 complexes of four Omicron subvariants (BA.1, BA.2, BA.3 and BA.4/5), and compared them with wild type complex (RBD_WT_-ACE2) in terms of various structural dynamic properties by molecular dynamics (MD) simulations and binding free energy (BFE) calculations. The results of MD simulations suggest that the RBDs of all the Omicron subvariants (RBD_OMI_s) feature increased global structural fluctuations when compared with RBD_WT_. Detailed comparison of BFE components reveals that the enhanced electrostatic attractive interactions are the main determinant of the higher ACE2-binding affinity of RBD_OMI_s than RBD_WT_, while the weakened electrostatic attractive interactions determine RBD of BA.4/5 subvariant (RBD_BA.4/5_) lowest ACE2-binding affinity among all Omicron subvariants. The per-residue BFE decompositions and the hydrogen bond (HB) networks analyses indicate that the enhanced electrostatic attractive interactions are mainly through gain/loss of the positively/negatively charged residues, and the formation or destruction of the interfacial HBs and salt bridges can also largely affect the ACE2-binding affinity of RBD. It is worth pointing out that since Q493R plays the most important positive contribution in enhancing binding affinity, the absence of this mutation in RBD_BA.4/5_ results in a significantly weaker binding affinity to ACE2 than other Omicron subvariants. Our results provide insight into the role of electrostatic interactions in determining of the binding affinity of SARS-CoV-2 RBD to human ACE2.

## 1. Introduction

Since December 2019, the world pandemic of the severe acute respiratory syndrome coronavirus 2 (SARS-CoV-2) has posed a serious threat to human health and world economy [1,2]. As of August 2022, SARS-CoV-2 has accounted for more than 590 million infections and more than six million deaths worldwide according to data released by the WHO (https://www.who.int/emergencies/diseases/novel-coronavirus-2019 (accessed on 29 August 2022)).

**Figure 1:**
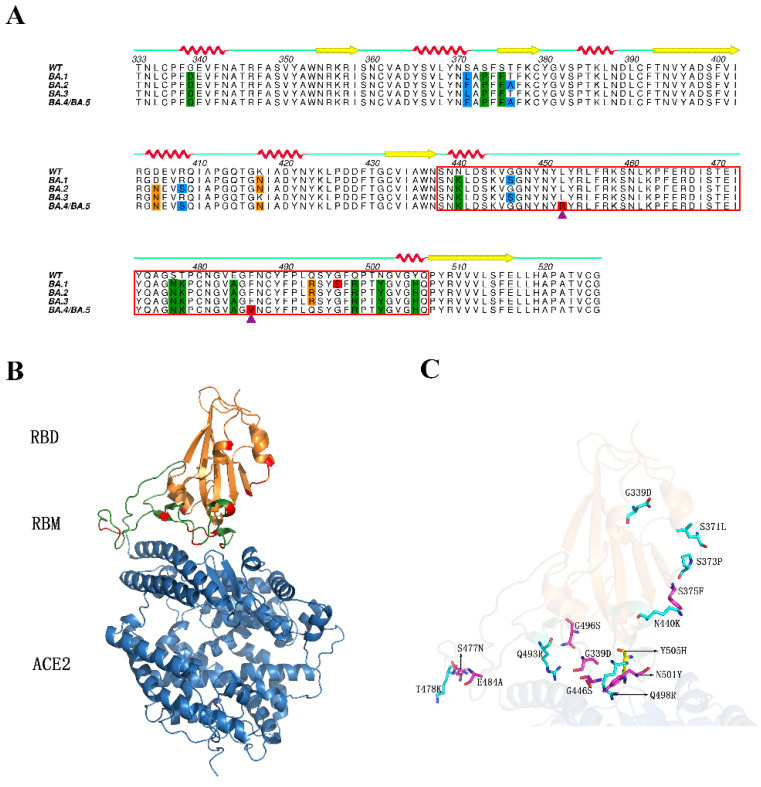
Multiple sequence alignment of RBD_WT_ and RBD_OMI_s, and 3D structures of RBD_BA.1_-ACE2 complex. (**A**) Multiple sequence alignment of RBD_WT_ and RBD_OMI_s. The residues with mutations existing in four, three, and two subvariants, and residues harboring unique mutation, are colored green, orange, blue and red, respectively. The L452R and F486V mutations, existing only in RBD_BA.4/5_, are indicated by purple triangles. RBM is highlighted by a red box. Secondary structures are illustrated above the corresponding amino acid sequence (red helix: α-helix; yellow arrow: β-sheet), and residue numbers are indicated above the primary sequence. (**B**) Ribbon representation of 3D structure of RBD_BA.1_-ACE2 complex (modeled on the crystal structure with PDB ID 6M0J [5]). ACE2 is colored blue. Core and RBM of RBD_BA.1_ are colored orange and green, respectively. The residues with mutations are highlighted in red. (**C**) Close-up view of RBD_BA.1_ bound to ACE2. The residues with mutations are represented as a stick model. (**A**) was generated using Aline [12], (**B**,**C**) were generated using Pymol [13].

Although both the entry of SARS-CoV-2 and SARS-CoV are triggered by the binding of the spike protein to angiotensin converting enzyme 2 (ACE2), the former is obviously more “cunning” than the latter and has evolved higher transmissibility [3,4,5]. The structure of the SARS-CoV-2 spike protein, which is composed of three identical protomers, has been described in detail in many studies [6,7]. Each subunit of the spike trimer consists of two large domains, namely, the N-terminal domain (NTD) and C-terminal domain (CTD). The CTD, which is also known as the receptor binding domain (RBD), plays a significant role in the recognition between spike and ACE2 (Figure 1B). The RBD can be further divided into a core region consisting of five antiparallel β-sheet and seven α-helix, and a receptor binding motif (RBM) (Figure 1B), which are located at the contact region of the spike and ACE2. Previous studies have shown that when compared with SARS-CoV, the changes of some key residues in the RBD of the SARS-CoV-2 spike protein can enhance its binding affinity to ACE2, which causes SARS-CoV-2 to be more infectious than SARS-CoV [3,8,9,10,11].

Due to the inherently high variability of RNA viruses, SARS-CoV-2 continuously evolves as changes in the genetic code occur during replication of the genome [14]. Since the first outbreak in Wuhan, Hubei Province of China, many clinically significant SARS-CoV-2 variants have emerged. The WHO has defined some specific variants as a ‘variant of concern (VOC)’, as the mutations they harbored could lead to their high transmissibility [15]. After Alpha (B.1.1.7), Beta (B.1.351), Gamma (P.1) and Delta (B.1.617.2) VOCs, the new Omicron variant (B.1.1.529), which was first reported to the WHO from South Africa on November 2021, has gradually become the predominant strain in the world [16,17]. The Omicron variant, similar to other variants, is comprised of a number of descendent lineages, including BA.1, BA.2, and BA.3, as well as now BA.4 and BA.5. Different from other VOCs, the Omicron variant has accumulated a huge amount of mutations, with more than 30 in the spike and about 10 in the RBD [18]. The multiple sequence alignment of the RBD of wild type (WT) SARS-CoV-2 (RBD_WT_) and RBDs of Omicron subvariants are shown in Figure 1A. It is worth mentioning that the BA.4 and BA.5 subvariants are often discussed together because the mutations in their spike are identical [19]. When compared with other Omicron subvariants, the BA.4 and BA.5 carry their own unique mutations in spike RBD (L452R and F486V). Currently, the BA.4 and BA.5 are expected to become the dominant SARS-CoV-2 strains since they are better able to evade immunity from vaccines and previous infections [20,21,22]. For the convenience of expression in this study, BA.4 and BA.5 are collectively referred to as BA.4/5 subvariant. The RBDs representing all the Omicron subvariants and the four individual subvariants are abbreviated as RBD_OMI_s, RBD_BA.1_, RBD_BA.2_, RBD_BA.3_, RBD_BA.4/5_, respectively.

Multiple previous studies show that the RBD_OMI_s have a higher binding affinity to ACE2 than that of RBD_WT_, which can provide a reasonable explanation for the high transmissibility of the Omicron variant [23,24,25,26,27,28,29,30]. However, differences in RBD-ACE2 binding affinity between the individual Omicron subvariants and the underlying mechanisms have not been well studied. Although the crystal structures of RBD_WT_ and RBD_OMI_s in complex with ACE2 have provided insight into the structural basis responsible for their different binding affinities [23,31,32,33,34], the thermodynamics and dynamics of RBD-ACE2 interactions still remain to be elucidated. An understanding of the mechanistic origin that determines the binding affinity of SARS-CoV-2 spike RBD to ACE2 is of great significance for variant detection, epidemic, and inhibitor design.

In this study, we performed molecular dynamics (MD) simulations on the constructed homology models of RBD_OMI_s-ACE2 complexes of four Omicron subvariants (BA.1, BA.2, BA.3 and BA.4/5), and compared them with RBD_WT_-ACE2 in terms of conformational flexibility, binding free energies (BFEs), buried solvent accessible surface areas (BSAs), electrostatic surface potentials (ESPs) and hydrogen-bonding networks (HBNs). Our study provides insights into the dominant role of electrostatic interactions in determining the binding affinity of SARS-CoV-2 spike RBD to ACE2 and sheds light on surveillance of new SARS-CoV-2 variants and RBD-ACE2 binding inhibitor design.

## 2. Results

### 2.1. Structural Stability and Flexibility during Simulations

**Figure 2:**
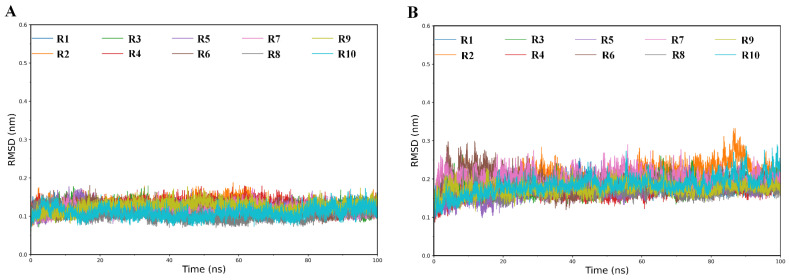
Time-dependent backbone RMSD values of RBD and ACE2 of WT complex relative to the starting structures calculated from the 10 independent replicas (R1–R10). (**A**) RBD; (**B**) ACE2.

The stability/equilibrium of our simulations were examined by calculating the time-dependent backbone root mean square deviation (RMSD) values for each replica of the simulation systems. The RMSD curves of RBD and ACE2 of WT complex are shown in Figure 2, and the RMSD curves of RBD and ACE2 of Omicron complex are shown in Figures S1 and S2, respectively. As shown in Figure 2, Figures S1 and S2, the RMSD curves of ACE2 (Figure 2B and Figure S2) have a relatively larger vibration amplitude than that of RBD (Figure 2A and Figure S1), indicating that the former experienced larger global structural fluctuations than the latter during the simulations. For all simulation systems, each of their replicas was used to calculate RMSF, BFE and other structural and geometrical properties, and further to obtain the average values.

**Figure 3:**
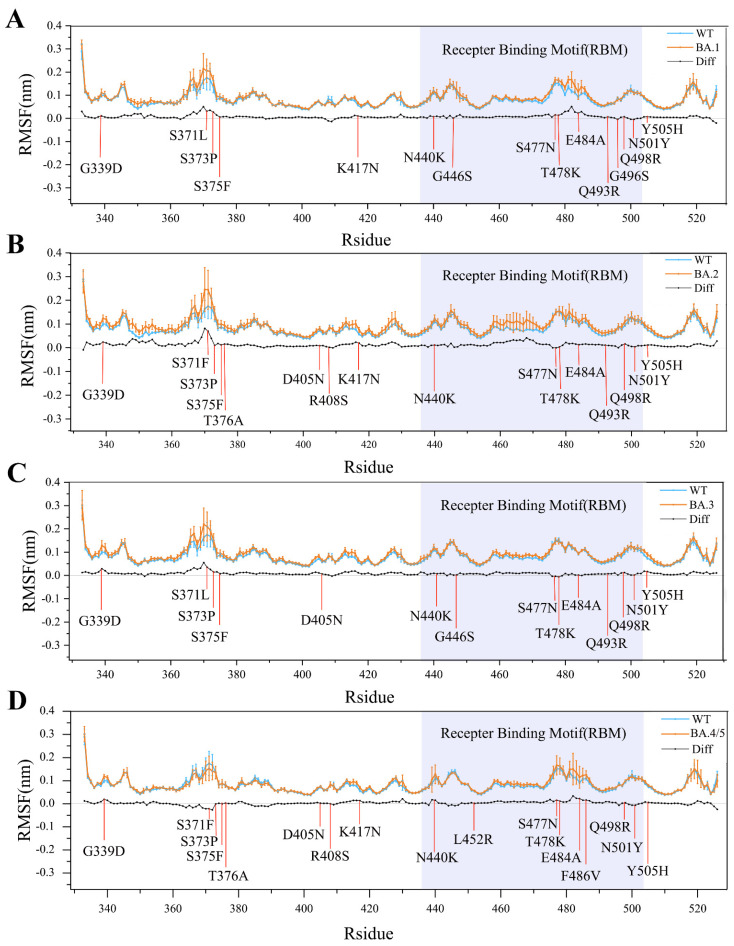
Comparison between the structural flexibility of RBD_OMI_s (orange line) and RBD_WT_ (blue line). (**A**) RBD_BA.1_ and RBD_WT_. (**B**) RBD_BA.2_ and RBD_WT_. (**C**) RBD_BA.3_ and RBD_WT_. (**D**) RBD_BA.4/5_ and RBD_WT_. The flexibility is measured by per-residue RMSF values with error bars of different replicas. The mutations in RBD_OMI_s are highlighted in red. RMSF-difference (black line) was obtained by subtracting RBD_WT_ RMSF values from RBD_OMI_s values. The RBM region is shaded in light blue.

In order to compare the structural flexibility of RBD_WT_ and RBD_OMI_s, we calculated the values of per-residue Cα atom RMSF and standard errors of different replicas during simulations (Figure 3). As shown in Figure 3, although the trends of all the RMSF curves are almost identical (e.g., the secondary structure elements and buried hydrophobic core exhibit low RMSF values and standard error, and the surface-exposed loops and N-, C- termini exhibit high RMSF values and standard error), the four RBD_OMI_s (RBD_BA.1_, RBD_BA.2_, RBD_BA.3_, RBD_BA.4/5_) have a relatively higher flexibility than RBD_WT_ in many structural regions. The increased overall flexibility or conformational freedom (decreased structural stability) could enable RBD_OMI_s with richer conformational diversity, thereby favoring their increased ACE2-binding affinity. Close examination of Figure 3 suggests that most of mutation sites of RBD_OMI_s show larger RMSF values or increased flexibility than RBD_WT_, indicating that these mutations may make positive contributions to enhancing the ACE2-binding affinity of RBD_OMI_s.

### 2.2. Binding Free Energy Calculation

In order to compare the ACE2-binding affinity of RBD_WT_ and the four RBD_OMI_s, we calculated the BFE values using the molecular mechanics Possion–Boltzmann surface area (MM-PBSA) algorithm. As presented in Table 1 and Table S1, the final BFE value (ΔG_binding_) of RBD_WT_-ACE2 complex is higher than all of the four Omicron subvariants, indicating that each RBD_OMI_s structure has higher ACE2-binding affinity than RBD_WT_. When comparing within the subvariants, the BFE values from high to low are: BA.4/5 (−51.67), BA.1 (−65.66), BA.2 (−70.44), and BA.3 (−79.78), indicating RBD_BA.3_ has the highest ACE2-binding affinity, while RBD_BA.4/5_ has the lowest. It is worth noting that the Omicron BA.4/5 has gradually replaced the previous subvariants as the predominant SARS-CoV-2 strain, therefore, our BFE results can suggest that the high transmissibility of BA.4/5 may primarily originate from the enhanced immune evasion rather than the higher ACE2-binding affinity.

Detailed decomposition of several BFE components revealed that the electrostatic interaction potential energy (ΔE_ele_) is the primary force driving the binding events for all the RBD-ACE2 complexes, followed by van der Waals (vdW) interaction potential energy (ΔE_vdw_) and non-polar solvation free energy (ΔG_nonpolar_). It is worth pointing out that since the ΔG_nonpolar_ term can represent the hydrophobic effect caused by the gain of solvent entropy upon binding, our BFE results revealed that the three commonly non-bonded attractive interactions (electrostatic, vdW and hydrophobic effect) are all enhanced in RBD_OMI_s-ACE2 compared with RBD_WT_-ACE2. In contrast, the polar solvation free energy (ΔG_polar_) negatively contributes to the binding events, and whose effects are mainly compensated by the ΔE_ele_ term. Close examination of Table 1 shows that, unlike the other two terms that play positive role(ΔE_vdw_ and ΔG_nonpolar_), the trend of ΔE_ele_ values is completely consistent with ΔG_bind_ term, indicating that electrostatic attractive interactions are the primary determinant of the higher ACE2-binding affinity of RBD_OMI_s than that of RBD_WT_.

**Figure 4:**
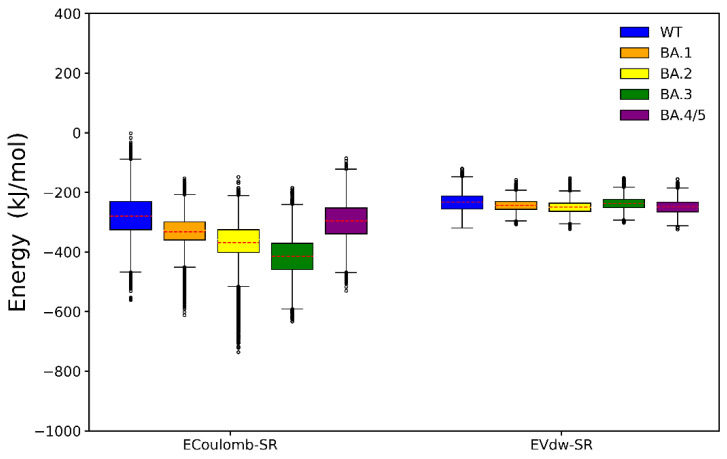
Boxplot of short-range electrostatic (Ecoulomb-SR) and van der Waals (Evdw-SR) interaction energies. The average values are highlighted in red dotted line.

To further investigate and compare the roles of electrostatic and vdW attractive interactions in determining the RBD-ACE2 binding affinities in different SARS-CoV-2 strains, we also calculated the potential energies between RBD and ACE2 during simulations (Figure 4). It is worth pointing out that since we only calculated short-range electrostatic interactions, Figure 4 cannot fully represent the overall electrostatic interactions. As shown in Figure 4, the short-range electrostatic attractive interactions are significantly stronger than vdW attractive interactions in all simulation systems, and the differences in the former are much larger than the latter. In addition, as for the ΔE_ele_ term in BFE calculation, the trend of short-range electrostatic attractive interactions is also very consistent with the RBD-ACE2 binding affinity, further indicating that electrostatic interactions are the primary determinant of the binding affinity of SARS-CoV-2 RBD to ACE2.

**Figure 5:**
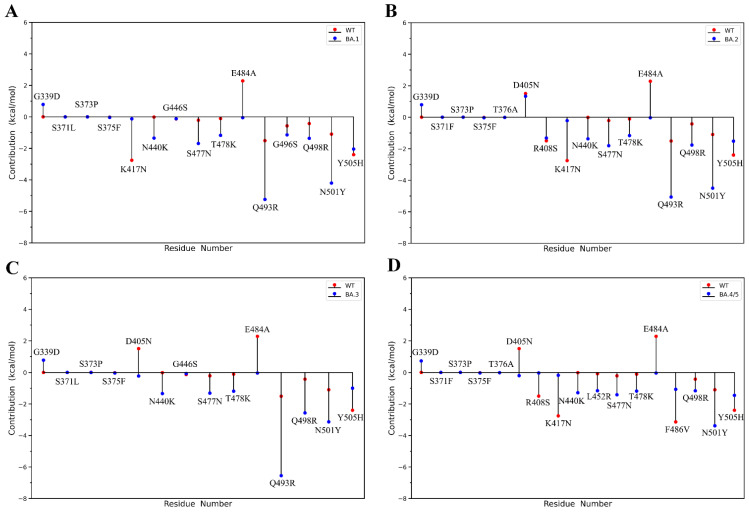
Per-residue contributions of each mutation site to binding free energy (BFE) values calculated using MM-PBSA method. (**A**) RBD_BA.1_ and RBD_WT_. (**B**) RBD_BA.2_ and RBD_WT_. (**C**) RBD_BA.3_ and RBD_WT_. (**D**) RBD_BA.4/5_ and RBD_WT_. Note: If the residue site with per-residue BFE value is lower in RBD_OMI_s than in RBD_WT_, it indicates that mutation at this site makes a positive contribution to enhancing the ACE2-binding affinity of RBD_OMI_s.

The per-residue contributions of each mutation site to BFE values were calculated and compared between RBD_WT_ and RBD_OMI_s (Figure 5). It is worth pointing out that if the per-residue BFE value is lower in RBD_OMI_s than that in RBD_WT_, it indicates that mutation at this site makes a positive contribution to enhancing the ACE2-binding affinity of RBD_OMI_s. As shown in Figure 5, mutations that can enhance ACE2-binding affinity are all distributed within the RBM, including D405N, N440K, L452R, S477N, T478K, E484A, Q493R, Q498R and N501Y, among which Q493R and N501Y are the most significant. Close examination of the charge properties of these mutations revealed that, with the exception of S477N and N501Y, the other residue changes are all involved in the charge changes (the loss of the negatively charged residue or the gain of the positively charged residue). Mutations that negatively contribute to ACE2-binding affinities include G339D, R408S, K417N, F486V, and Y505H, and in contrast to the above mutations that play a positive role, the charge changes of the first three of them are the gain of negative charge and the loss of positive charge, respectively. In summary, our per-residue BFE calculations revealed that the increasing of the positive electrostatic potential of RBM is significantly positively correlated with the enhanced ACE2-binding affinities of RBD_OMI_s, which further confirms the crucial role of electrostatic interactions in determining the binding affinity of SARS-CoV-2 spike RBD to ACE2.

### 2.3. Analyses of Interfacial Interactions bwtween RBD and ACE2

In order to further interpret the results of BFE calculations and explore the structural origin that determines RBD-ACE2 binding affinity, we compared the structure features and intermolecular non-bonded interactions at the ACE2-RBD interface between RBD_OMI_s-ACE2 and RBD_WT_-ACE2 by performing the dynamic structural and geometrical properties analyses. We first calculated the number of residue–residue contacts between RBD and ACE2 using the contact map strategy. As shown in Tables S2–S6, the contact numbers do not show a clear trend between different virus strains.

**Figure 6:**
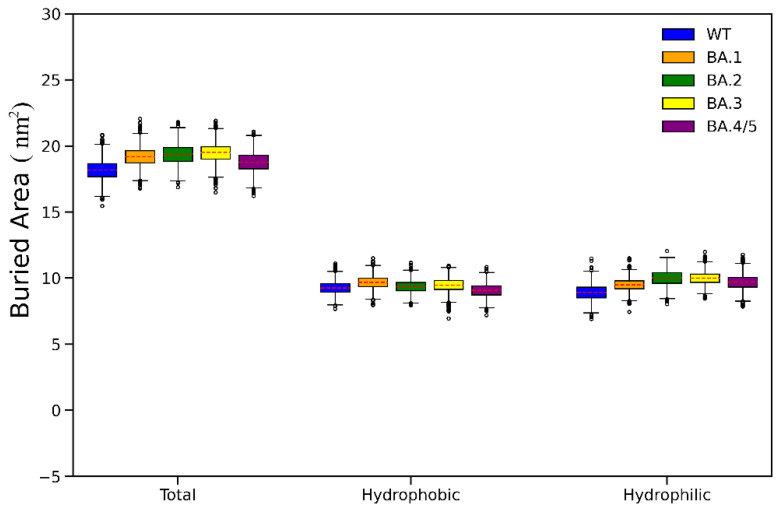
Boxplot of the buried total, hydrophobic and hydrophilic binding surface areas (BSAs) of the RBD-ACE2 binding interface. The average values are highlighted in red dotted line.

The BSA is a commonly used indicator to evaluate the size of the interface between two macromolecules in a protein–protein complex. Since sufficient contact area is the prerequisite for protein–protein recognitions, the value of BSA can reflect the strength of the overall intermolecular interactions to a certain extent. In this study, the buried total, hydrophobic and hydrophilic BSAs upon RBD-ACE2 bindings were calculated and used to highlight or reflect the strength of vdW interactions, hydrophobic effects and electrostatic interactions, respectively. As shown in Figure 6, almost all average values (highlighted in red dotted line) of the BSAs in RBD_OMI_s-ACE2 complexes are higher than those in RBD_WT_-ACE2, thus, revealing that the three non-bonded interactions are more enhanced in RBD_OMI_s-ACE2 than in RBD_WT_-ACE2. When comparing within the Omicron subvariants, the trends of the BSAs are consistent with the BFEs, thus, providing another indication that RBD_BA.4/5_ has the lowest ACE2-binding affinity among all RBD_OMI_s. Notably, the differences in total and hydrophilic BSAs between the SARS-CoV-2 variants are significantly higher than that of hydrophobic BSAs, which can explain the result of decomposition of BFE components, that the hydrophobic effect is the weakest force driving the binding of RBD and ACE2.

**Figure 7:**
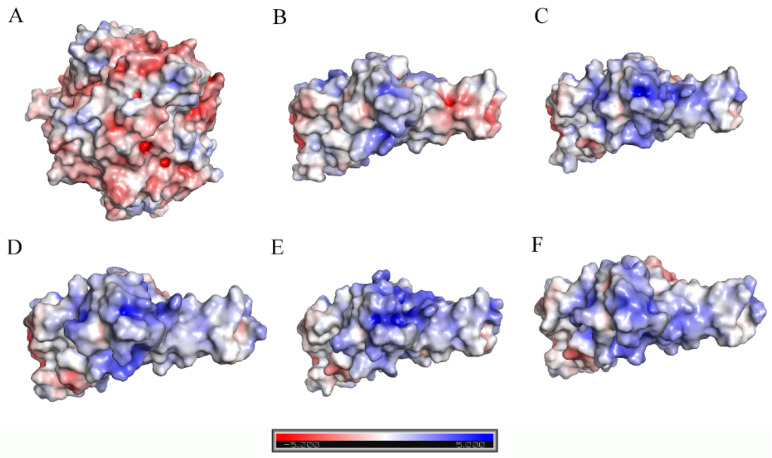
The electrostatic surface potentials (ESPs) of the binding surface of ACE2 and RBDs. (**A**) ACE2. (**B**) RBD_WT_. (**C**) RBD_BA.1_. (**D**) RBD_BA.2_. (**E**) RBD_BA.3_. (**F**) RBD_BA.4/5_. The red and blue colors denote the negative and positive potentials, respectively.

The electrostatic attractive interactions between RBD and ACE2 were further depicted or reflected by constructing their interfacial ESPs. As shown in Figure 7, the interfacial ESPs of ACE2 and all the RBD molecules have large negative and positive (or neutral) charged regions, respectively, which indicates that the electrostatic attractive interactions are crucial in driving the binding of RBD and ACE2. However, the size of positive charge regions in RBD_OMI_s are significantly larger compared with RBD_WT_, explaining why the electrostatic attractive forces (ΔE_ele_ term of BFE) are stronger in RBD_OMI_s-ACE2 than in RBD_WT_-ACE2. Notably, due to the absence of Q493R mutations, RBD_BA.4/5_ has the smallest positive region of all the RBD_OMI_s, which is consistent with the BFE conclusion that it has the lowest ACE2-binding affinity among the four RBD_OMI_s. In summary, the comparative analyses of ESPs can provide a plausible explanation for why the electrostatic interactions are the primary determinant for the binding affinity of RBD to ACE2, and why the loss of the negatively-charged residue or the gain of the positively-charged residue can enhance the RBD-ACE2 binding affinity.

**Figure 8:**
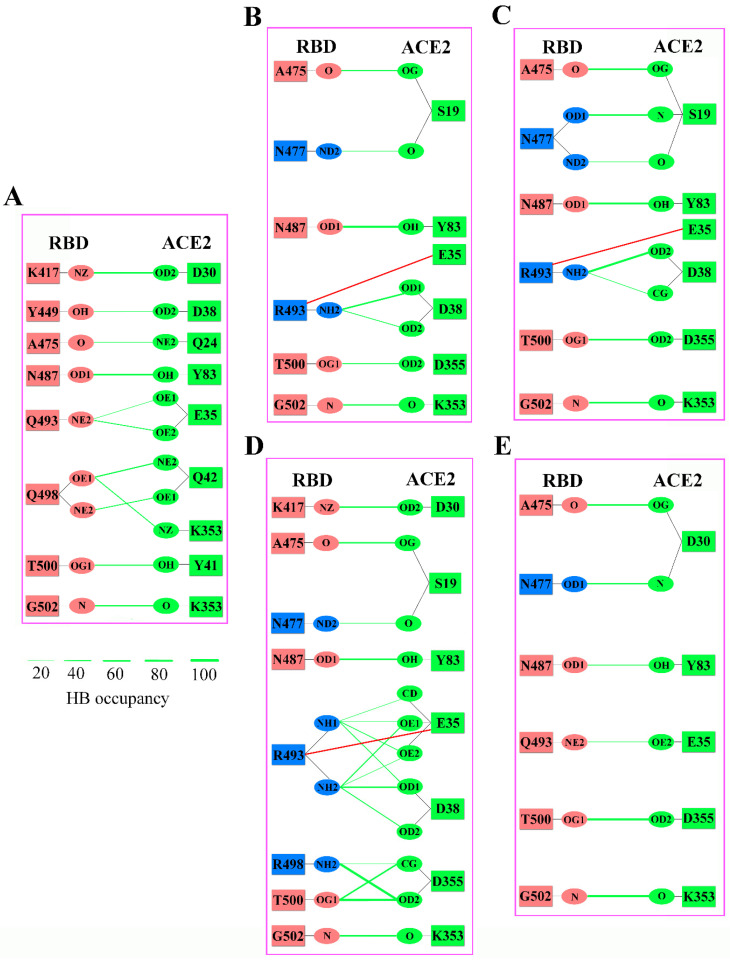
The hydrogen bond networks (HBNs) and salt bridges (SBs) across the binding interfaces of the RBD_WT_-ACE2 and RBD_OMI_s-ACE2 complexes. (**A**) RBD_WT_-ACE2. (**B**) RBD_BA.1_-ACE2. (**C**) RBD_BA.2_-ACE2. (**D**) RBD_BA.3_-ACE2. (**E**) RBD_BA.4/5_-ACE2. The RBD WT residues are colored in deep salmon, RBD mutated residues are colored in light blue, ACE2 residues are colored in green. The HBs with an occupancy greater than 20% are shown as green lines, SBs are represented as red lines, respectively.

As the two important short-range electrostatic attractive interactions, hydrogen bond (HB) and salt bridge (SB) play a crucial role in stabilizing the protein–protein complexes. As shown in Figure 8, with the exception of RBD_BA.3_-ACE2, the numbers of interfacial HBs in other RBD_OMI_s-ACE2 complexes are smaller than in RBD_WT_-ACE2, indicating that the enhanced electrostatic attractive interactions of the former are mainly due to the increase in the number of residue charge changes (the loss of the negatively-charged residue or the gain of the positively-charged residue) which can increase the positive potential of RBM and the ionic attractive interactions between RBD and ACE2, rather than the increase in the number of HBs. However, the loss/gain of HBs can still make significant negative/positive contributions to the RBD-ACE2 binding affinity. For example, although no charge change is involved, the S477N mutation still enhances the ACE2-binding affinity by about −1.5 to −2 kcal/mol (Figure 5) through forming a new HB between the mutated residue N477 and ACE2 residue S19, and this range of values is roughly comparable to BFE changes due to mutations involving only charge changes (D405N, N440K, T478K, E484A and Q498R). In addition to the above mutations with only one kind of change, the K417N mutation involves both HB and charge changes. For the K417N mutation, the loss of one positive charge and one HB (formed between RBD residue K417 and ACE2 residue D30) increases BFE by about 2 to 2.5 kcal/mol (Figure 5), thus, explaining why it makes the largest negative contribution to the binding affinity of RBD and ACE2. On the contrary, the Q493R mutation makes the most significant positive contribution by reducing BFE by about −3 to −6 kcal/mol, as it not only retains and strengthens the HBs formed between RBD WT residue Q493 and ACE2, but also forms a new SB between mutated residue R493 and ACE2 residue E35. Close examination of Figure 8 suggests that although the WT and mutated residues are identical in RBD 493 and 498 sites, the Q498R mutation makes a significantly smaller contribution to enhancing the binding affinity of RBD_OMI_s to ACE2 due to the absence of SBs and HBs.

In summary, analyses of interfacial interactions between RBD and ACE2 reveal that: (i) the enhanced overall non-bonded intermolecular interactions of RBD-ACE2 complexes primarily originate from the larger interfacial BSAs, (ii) the increase in positive ESPs of RBM enhance the electrostatic attractive interactions and binding affinity between RBD and ACE2, (iii) SB plays a greater role in enhancing the RBD-ACE2 binding affinity than HB and the general ionic attractive interaction, and, (iv) due to the absence of an SB caused by Q493R mutation, the RBD_BA.4/5_ has the weakest ACE2-binding affinity among all RBD_OMI_s, although the increased general ionic attractive interaction still makes it stronger than RBD_WT_.

## 3. Discussions

Although vaccines have been approved and widely distributed in various countries, SARS-CoV-2 is still spreading rapidly around the world due to the high mutation rate of the viral genome [35]. Currently, the Omicron BA.4 and BA.5 have replaced BA.2 as the dominant SARS-CoV-2 strains due to their high transmissibility [36]. The high transmissibility of the new SARS-CoV-2 variants may be attributed to its spike RBD being more likely to be “up” state, which can facilitate the binding (or interaction) of spike and ACE2 [8,37]. For example, a recently study by Sztain et al. showed that the glycosylation of several residues of RBD can facilitate RBD opening and to be “up” state [38]. On the basis that RBD is in the “up” state, its ACE2-binding affinity further affects SARS-CoV-2 transmissibility. Koehler et al. [39] provided thermodynamic and kinetic insight into the binding of several VOCs RBDs and ACE2. Their results showed that the RBDs of four VOCs (Alpha-RBD, Beta-RBD, Gamma-RBD, Kappa-RBD ) have higher ACE2-binding affinity than WT. Other experimental and structural studies covering Omicron and Delta VOCs indicated that Omicron RBD has a relatively higher ACE2-binding affinity than WT, but slightly lower than Delta [33]. Although comparison of crystal and cryo-EM structures provided valuable insights [33,34], the underlying mechanics and energetics (thermodynamics) mechanisms modulating the RBD-ACE2 binding affinity need to be elucidated by dynamic studies.

Structural quality evaluation by PROCHECK 3.5 shows that for both of our constructed models, more than 90% of residues fall within the favored/allowed regions of the Ramachandran plots (Figures S3–S7), indicating that they are suitable for characterizing the changes in structure and dynamics of RBD-ACE2 complexes upon mutations. The MD simulations of the models reveal that, when compared with RBD_WT_, the four RBD_OMI_s feature increased overall structural flexibility. Based on conformational selection theory [40,41], the higher flexibility is an important prerequisite for a protein to undergo various conformations, which could favor it to recognize and bind the ligand. Similar conclusions were also obtained in previous comparative studies on RBD-ACE2 binding affinity of SARS-CoV-2 and SARS-CoV, which indicated that the RBD of the former showed higher global structural fluctuation than that of the latter. In addition to the positive correlation between the overall structural fluctuation and ACE2-binding affinity, we found that the fluctuation at the single residue mutation site was also positively correlated with its contribution to binding affinity, which means that the greater the positive contribution of the mutation to ACE2-binding affinity, the higher fluctuation the residue site has. Therefore, the structural fluctuation can be used as an indicator to reflect the ACE2-binding affinity and the per-residue (mutation) contributions.

The comparison between BFEs of RBD_WT_-ACE2 and RBD_OMI_s-ACE2 complexes indicates that all RBD_OMI_s have a more enhanced ACE2-binding affinity than RBD_WT_, which is consistent with the former studies [28,29,30]. It is worth pointing out that all Omicron variants have lager BSAs at the RBD-ACE2 interface than WT, providing a positive correlation between enhanced BFE and larger BSA. Our results about the relationship between BFE and BSA are consistent with those of Lupala et al. [25]. However, the binding affinity of RBD_BA.4/5_-ACE2 is not only not the highest as expected, but one of the lowest among all RBD_OMI_s. In contrast, although the RBD_BA.3_ has the highest ACE2-binding affinity, it did not become the dominant strain, and is even very rare in the world. The seemingly contradictory result shows that, in addition to ACE2-binding affinity, the immune evasion is also crucial for determining the SARS-CoV-2 transmissibility. Coincidentally, two recent studies [20,21] showed that the Omicron BA.4 and BA.5 were over four times more resistant to antibodies from vaccinated and boosted individuals than earlier strains of Omicron, indicating that they have had a very strong immune evasion due to the unique L452R and F486V mutations they have. Combined with our BFEs calculations and the above immune evasion study, it indicates that the ACE2-binding affinity and immune evasion of RBD together determine viral transmissibility. As the BA.4 and BA.5 continue to spread around the world, their immune evasion may diminish as the infection numbers increase and new vaccines are used. Coupled with their originally weaker ACE2-binding affinity, we can infer that the transmissibility of Omicron strain will gradually decrease over time. Nevertheless, since new SARS-CoV-2 variants may emerge in the near future, it is crucial to be able to monitor the effect of mutations on immune evasion and ACE2-binding affinity. Although the immune evasion is difficult to be effectively evaluated due to the variety of antibodies and binding sites, we can easily monitor and predict the changes of ACE2-binding affinity caused by mutations by understanding the mechanics and energetics (thermodynamics) mechanisms modulating the RBD-ACE2 binding affinity.

In order to achieve the above purpose, a detailed comparison of various non-bonded interactions in different SARS-CoV-2 variants should be performed. Although there were many related studies before the emergence of Omicron [42,43,44,45,46], these results may not necessarily reveal the predominant energetic determinants of ACE2-binding affinity due to too few mutations in previous VOCs (Alpha, Beta, Gamma and Delta). However, Omicron can be used as appropriate research material due to its large number of mutation sites and subvariants (BA.1, BA.2, BA.3, BA.4 and BA.5). The detailed comparison of various BFE terms and short-range interaction energies of RBD_WT_-ACE2 and RBD_OMI_s-ACE2 complexes indicates that, when compared with vdW interactions and hydrophobic effects, the electrostatic attractive interactions are the primary determinant of the enhanced binding affinity of RBD to ACE2. In addition, when comparing SARS-CoV-2 and SARS-CoV, the identical conclusion was obtained, that is, the electrostatic attractive interactions primarily determine the higher ACE2-binding affinity of RBD of the former, than the latter [8]. Although SARS-CoV-2 is unlikely to have evolved directly from SARS-CoV, we can also infer that it may be because the previous generation of SARS-CoV-2 acquired mutations that significantly enhanced its electrostatic attractive interactions with human ACE2 to the point that it finally evolved the ability to infect humans. It is worth pointing out that although van der Waals forces are also driven by electrical interactions between neutral atoms or molecules, the term “electrostatic interaction” in this study refers only to the direct ionic interactions (including SBs) and HBs.

The overall ionic attractive interactions between RBD and ACE2 can be attributed to the fact that their own binding interface has large positive and negative ESPs, respectively, which explains why the electrostatic attractive interactions are crucial for the enhanced binding affinity of RBD to ACE2. Therefore, as depicted by per-residue decomposition of BFEs, most of the mutations that make significantly positive or negative contributions to ACE2-binding affinity involve the charge changes. For example, mutations with loss of negative charge or gain of positive charge positively contribute to ACE-binding affinity, and vice versa. In addition to changes involving integer charges, partial charges are also critical for RBD-ACE2 binding. Podgornik et al. showed that mutations in the Omicron RBD were connected with a significant increase in the positive partial charge, which facilitates the interaction with ACE2 [47].

However, the Y505H is the only exception. Although the Y505H mutation imparts a positive charge to RBD, it makes a negative contribution to the ACE-binding affinity. How can this seemingly opposite result be explained? We infer the reason is that, as being an aromatic amino acid, Y505 residue has already formed stable π–π interactions with the ACE2 molecule, which make a relatively larger positive contribution to the binding affinity of RBD to ACE2 by about −2 kcal/mol (Figure 5). When the Y505 residue was mutated to histidine, the positive contribution due to gaining a positive charge (about −1.5 kcal/mol) did not sufficiently compensate the negative contribution due to the loss of the π–π interaction. The effect of the π–π interaction to the ACE2-binding affinity can also be demonstrated by the example of the N501Y mutation [42], where mutation of N501 residue to tyrosine decreased the BFE of the RBD-ACE complex by about −4 kcal/mol (Figure 5) in our study. Of note is that, although the positive contribution of the π–π interaction mediated by a single residue to ACE2-binding affinity may be greater than that of an increase in general ionic attractive interaction due to charge change, the electrostatic interactions are still the primary determinant of the binding affinity of RBD to ACE2 due to less aromatic amino acids and intermolecular π–π interactions in the RBD-ACE2 complex.

Apart from the general ionic attractive interactions, HBs and SBs also play crucial roles in enhancing the binding affinity of RBD-ACE2 complexes. For example, since the RBD WT residue K417 forms a strong HB with ACE2 residue D30 (Figure 8), the simultaneous loss of a positive charge and a HB cause K417N to make a significantly larger negative contribution to ACE2-binding affinity than other mutations involving only positive charge loss or negative charge gain. In contrast, the simultaneous gain of a positive charge and an SB cause Q493R to make the largest positive contribution among all mutations. Therefore, the ionic attractive interactions (including SBs) and HBs collectively enhance, although to different extents, the ACE2-binding affinity of RBD. It is worth pointing out that absence of K417N in RBD_BA.3_ and Q493R in RBD_BA.4/5_ can explain why they have the strongest and weakest binding affinity to ACE2 (Table 1), respectively.

In summary, our study concludes that the electrostatic interactions are the primary determinant of the binding affinity of the SARS-CoV-2 spike RBD to ACE2. In addition to the general ionic interaction changes caused by the gain/loss of the positively/negatively charged residues, the formation or destruction of the interfacial HBs and SBs can also largely affect the ACE2-binding affinity of RBD.

## 4. Materials and Methods

### 4.1. Structure Preparation

The X-ray crystallographic structure of the RBD_WT_-ACE2 complex was obtained from the Protein Data Bank (www.pdb.org (accessed on 16 February 2022)) with PDB ID 6M0J [5]. The RBD_OMI_s homology models of the five Omicron subvariants were built with MODELLER 10.0 software package [48] using RBD_WT_ as a template. The structures of the RBD_OMI_s and ACE2 complexes were obtained by superposing the homology models of RBD_OMI_s to the X-ray structure of the RBD_WT_-ACE2 complex. The Ramachandran plots of the complexes were generated by PROCHECK [49] module in SAVES (https://saves.mbi.ucla.edu (accessed on 10 November 2022)).

### 4.2. MD Simulation

All simulations were performed using the GROMACS2020.6 software package [50] with the AMBER99SB forcefield [51]. Each complex structure was protonated according to the protonation states of all titratable residues at pH 7.4. The protonated structures were dissolved using the TIP3P water model [52] and put in a dodecahedron box with the distance between any atom of the protein and the box wall being greater than 1.0 nm. The net charges of both simulation systems were neutralized with a 100 mM concentration of NaCl to mimic the physiological conditions (number of net charges and several other quantities in the setup are shown in Table S7). The simulation systems were firstly subjected to energy minimization using the steepest descent algorithm, and then equilibrated by two continuous 10 ns position restraint simulations in the NVT and NPT ensembles with harmonic force constants of 1000 kJ·mol^−1^·nm^−2^. To improve the sampling of the conformational space and avoid false–positive conclusions in molecular simulation [53], each system was subjected to 10 independent 100-ns production MD simulations, with each replica initialized with different initial atomic velocities assigned from a Maxwell distribution at 310 K. The total simulation time for each complex was 1 μs.

The parameters and conditions of the production MD runs were: the LINCS algorithms [54] were used to constrain the bond lengths with the integration time step of 2 fs; the long-range electrostatic interactions were calculated by the particle-mesh Ewald (PME) algorithm [55] with interpolation order of 4, Fourier grid spacing of 0.135 nm and Coulomb radius of 1.0 nm; the van der Waals (VDW) interactions were modeled by Verlet scheme with a cut-off distance of 1 nm; the v-rescale thermostat [56] was used to couple the temperature of the systems at 310 K with a 0.1 ps time constant; the Parrinello–Rahman barostat [57] was used to maintain the pressure of the systems at 1 atm with a 0.5 ps time constant; and structural snapshots were saved every 10 ps.

### 4.3. Structural and Geometrical Properties

The following GROMACS tools were used to perform structural and geometrical analyses of MD trajectories: ‘gmx rms’ to calculate the time-dependent backbone root mean square deviation (RMSD) relative to the starting structure; ‘gmx rmsf’ to calculate the per-residue Cα root mean square fluctuation (RMSF); ‘gmx sasa’ to calculate the solvent accessible surface area (SASA); ‘gmx energy’ to calculate the short-range electrostatic and van der Waals interaction energy.

The hydrogen bonds formed between RBD-ACE2 were calculated by ‘Hydrogen Bonds’ plugin in VMD [58] with the donor–acceptor distance less than 3.5 Å and the donor-acceptor angle larger than 120°. The electrostatic surface potential of the structures of ACE2 and RBD were generated using Pymol 2.5.2 [13].

The binding of RBD and ACE2 will bury part of the molecular surface of the two molecules. The buried solvent accessible surface area (SASA) (*Area*_burial_) was calculated using the following equation:

*Area*_burial_ = (*SASA*_ACE2_ + *SASA*_RBD_) − *SASA*_complex_

where *SASA*_ACE2_, *SASA*_RBD_ and *SASA*_complex_ represent the SASA of ACE2, RBD and ACE2-RBD complex, respectively.

The residue–residue contacts were obtained by the GoContactMap online server (http://pomalab.ippt.pan.pl/GoContactMap/ (accessed on 10 November 2022)) [59,60]. The parameters used in GoContactMap were: the sequence distance was 4; the cutoff long was 1.1 nm; the cutoff short was 0.3 nm. In GoContactMap, the cutoff is dubbed or combined with a chemical-based algorithm, such as sphere overlap (OV) and repulsive contacts of structural units (rCSU).

### 4.4. Binding Free Energy (BFE) Calculation

Although the free-energy perturbation (FEP), which can estimate the difference in free energy between two states by slowly changing one state to another through a number of nonphysical intermediate states, is the most rigorous BFE computational method, this method is extremely time consuming. The molecular mechanics Poisson–Boltzmann surface area (MM/PBSA) and molecular mechanics generalized Born surface area (MM/GBSA) are the most commonly used approximate methods for calculating BFE [61]. In this study, the MM-PBSA algorithm [62] was used to calculate the BFE between ACE2 and RBD. MM-PBSA is a well-known endpoint approach which can estimate the protein-ligand BFE based merely on the structure or structural ensemble of the bound complex without considering either the physical or the non-physical intermediates [8,63,64]. In MM-PBSA, the BFE of the protein and ligand is defined as:

Δ*G*_binding_ = Δ*G*_complex_ − (Δ*G*_protein_ + Δ*G*_ligand_)

The free energy, *G*, of each subunit can be presented as:

*G* = *E*_MM_ +*G*_sol_ − *TS*

where *E*_MM_ represents the average molecular mechanical potential energy in vacuum, which is composed of the electrostatic (*E*_ele_) and van der Waals (*E*_vdw_) interactions and is presented as:

*E*_MM_ = *E*_ele_ + *E*_vdw_

*G*_sol_ represents the solvation free energy, which is decomposed into the polar (*G*_polar_) and non-polar (*G*_nonpolar_) components and is presented as:

*G*_sol_= *G*_polar_ + *G*_nonpolar_

*TS* represents the entropy contribution to the free energy, T represents temperature and S represents entropy. It is worth mentioning that since the TS component is negligible when comparing the relative BFEs between different systems and the calculation is challenging and time-consuming [62,65], the contribution of entropy was not included in our study.

The single trajectory approach implemented in gmx_MMPBSA 1.5.7 [66] was used to perform the MM-PBSA calculation. For each simulation system, the BFE between ACE2 and RBD was calculated for 100 snapshots of each replica using default parameters in gmx_MMPBSA. 

## Figures and Tables

**Table 1:** BFE values calculated by MM-PBSA method ^a^.

Energy Components	WT (kcal/mol)	Omicron BA.1 (kcal/mol)	Omicron BA.2 (kcal/mol)	Omicron BA.3 (kcal/mol)	Omicron BA.4/5 (kcal/mol)
ΔE_ele_	−615.20 (45.48)	−1368.55 (47.30)	−1381.35 (46.96)	−1948.76 (60.03)	−1285.69 (49.20)
ΔE_vdw_	−75.89 (6.53)	−81.71 (5.46)	−80.62 (5.50)	−78.74 (6.03)	−75.89 (5.04)
ΔG_MM_	−691.09 (46.95)	−1450.26 (47.39)	−1461.97 (46.85)	−2027.49 (59.95)	−1361.59 (50.12)
ΔG_polar_	655.95 (42.75)	1393.51 (45.39)	1402.02 (44.98)	1958.08 (57.46)	1319.13 (49.19)
ΔG_nonpolar_	−9.45 (0.58)	−9.80 (0.40)	−9.70 (0.38)	−10.37 (0.45)	−9.21 (0.45)
ΔG_sol_	646.50 (42.47)	1383.71 (45.26)	1391.53 (44.88)	1947.71 (57.69)	1309.92 (49.01)
ΔG_bind_	−44.59 (10.69)	−66.55 (9.24)	−70.44 (9.33)	−79.78 (10.11)	−51.67 (9.38)

^a^ Notes: ΔG_binding_ = E_MM_ + G_sol_; E_MM_ = E_ele_ + E_vdw_; G_sol_ = G_polar_ + G_nonpolar_; standard errors of different replicas of each simulation system are shown in brackets.

## Data Availability

All data are contained within the article or its supplementary materials as Figures or Tables.

## Supplementary Materials

The following supporting information can be downloaded at: https://www.mdpi.com/article/10.3390/ijms232314796/s1, Table S1. The calculated binding free energy components (kcal/mol)of each replica (1–10) of each simulation system; Table S2. Residue contacts between RBDWT and ACE2; Table S3. Residue contacts between RBDBA.1 and ACE2; Table S4. Residue contacts between RBDBA.2 and ACE2; Table S5. Residue contacts between RBDBA.3 and ACE2; Table S6. Residue contacts between RBDBA.4/5 and ACE2; Table S7. Several quantity information in MD simulation setup; Figure S1. Time-dependent backbone RMSD values of RBDOMIs relative to the starting structures calculated from the 10 independent MD simulations replicas (R1–R10); Figure S2. Time-dependent backbone RMSD values of ACE2 of RBDOMIs-ACE2 complexes relative to the starting structures calculated from the 10 independent MD simulations replicas (R1–R10); Figure S3. Ramachandran plot of RBDWT-ACE2 complex structure; Figure S4. Ramachandran plot of RBDBA.1-ACE2 complex structure; Figure S5. Ramachandran plot of RBDBA.2-ACE2 complex structure; Figure S6. Ramachandran plot of RBDBA.3-ACE2 complex structure; Figure S7. Ramachandran plot of RBDBA.4/5-ACE2 complex structure.
