# Supplementary material for: Electrostatic Interactions Are the Primary Determinant of the Binding Affinity of SARS-CoV-2 Spike RBD to ACE2: A Computational Case Study of Omicron Variants
Source: Int J Mol Sci. 2022 Nov 26;23(23):14796. doi: 10.3390/ijms232314796 (PMC9740405; doi:10.3390/ijms232314796)
Supplement: Supplementary file 1 [file ijms-23-14796-s001.zip › ijms-1919446-supplementary.pdf]

# Electrostatic Interactions Are the Primary Determinant of the Binding Affinity of SARS-CoV-2 Spike RBD to ACE2: A Computational Case Study of Omicron Variants

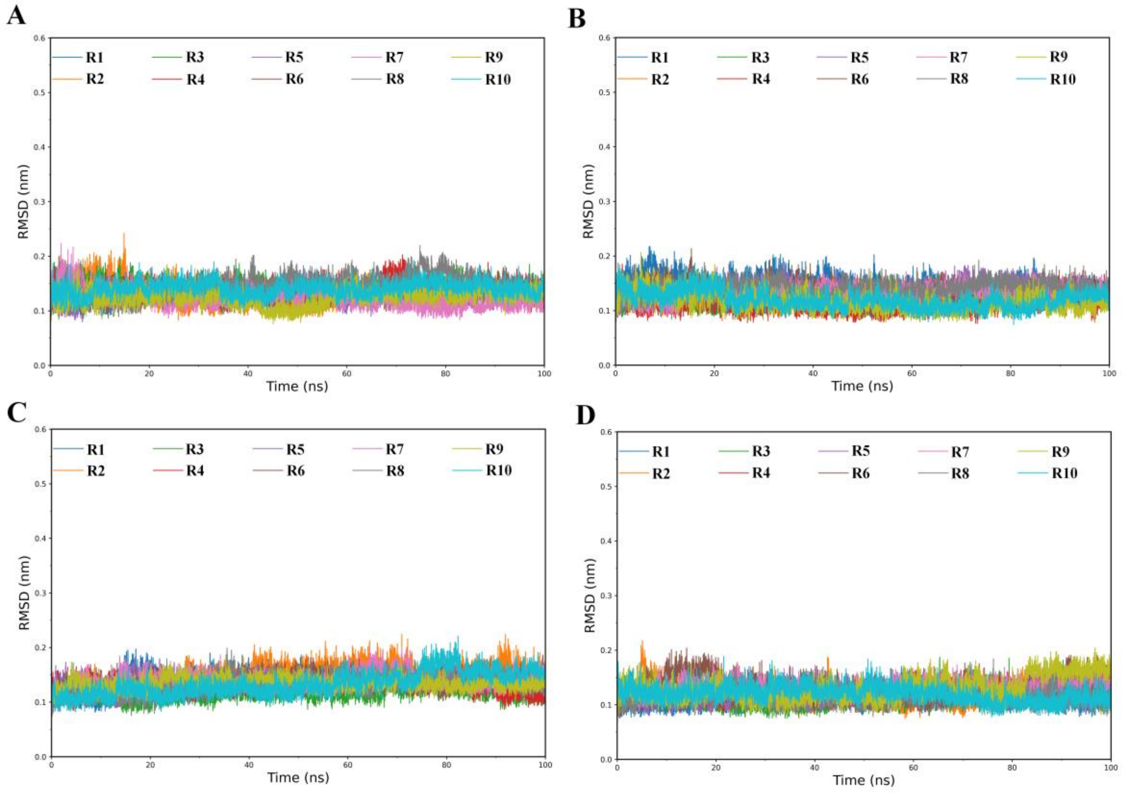


**Figure S1.** Time-dependent backbone RMSD values of $\mathrm{RBD_{OMIS}}$ relative to the starting structures calculated from the 10 independent MD simulations replicas (R1-R10). (**A**) $\mathrm{RBD_{BA.1}}$. (**B**) $\mathrm{RBD_{BA.2}}$. (**C**) $\mathrm{RBD_{BA.3}}$. (**D**) $\mathrm{RBD_{BA.4/5}}$.

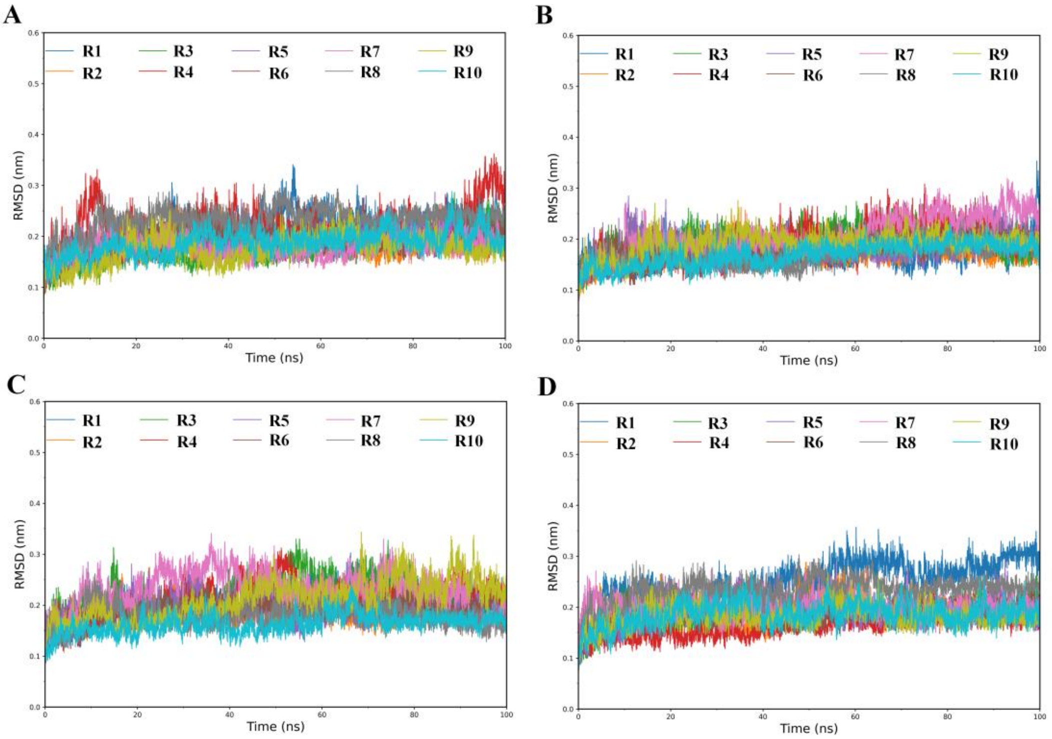


**Figure S2.** Time-dependent backbone RMSD values of ACE2 of RBD$_{\text{OMIS}}$-ACE2 complexes relative to the starting structures calculated from the 10 independent MD simulations replicas (R1-R10). (**A**) ACE2$_{\text{BA.1}}$. (**B**) ACE2$_{\text{BA.2}}$. (**C**) ACE2$_{\text{BA.3}}$. (**D**) ACE2$_{\text{BA.4/5}}$.

**Table S1. The calculated binding free energy components (kcal/mol)of each replica (1-10) of each simulation system**

| WT | 1 | 2 | 3 | 4 | 5 | 6 | 7 | 8 | 9 | 10 |
|---|---|---|---|---|---|---|---|---|---|---|
| $\Delta G_{complex}$ | -11067.12 | -11068.80 | -11077.98 | -11059.48 | -11109.32 | -11082.38 | -11052.48 | -11051.73 | -11063.46 | -11053.56 |
| $\Delta G_{ACE2}$ | -8600.64 | -8603.01 | -8604.08 | -8604.71 | -8639.70 | -8609.97 | -8567.48 | -8584.44 | -8588.56 | -8575.66 |
| $\Delta G_{RBD}$ | -2417.28 | -2430.00 | -2432.32 | -2413.96 | -2423.75 | -2432.16 | -2439.26 | -2424.48 | -2423.23 | -2425.77 |
| $\Delta G_{binding}$ | -49.20 | -35.80 | -41.58 | -40.81 | -45.87 | -40.25 | -45.74 | -42.81 | -51.67 | -52.13 |

| BA.1 | 1 | 2 | 3 | 4 | 5 | 6 | 7 | 8 | 9 | 10 |
|---|---|---|---|---|---|---|---|---|---|---|
| $\Delta G_{complex}$ | -11474.86 | -11513.08 | -11485.19 | -11463.29 | -11540.17 | -11471.95 | -11483.84 | -11463.42 | -11506.96 | -11502.85 |
| $\Delta G_{ACE2}$ | -8560.62 | -8610.19 | -8578.34 | -8565.63 | -8626.57 | -8578.18 | -8593.57 | -8569.45 | -8601.93 | -8586.26 |
| $\Delta G_{RBD}$ | -2845.16 | -2838.74 | -2837.33 | -2832.47 | -2848.65 | -2827.49 | -2824.28 | -2825.98 | -2842.48 | -2846.79 |
| $\Delta G_{binding}$ | -69.08 | -64.15 | -69.52 | -65.19 | -64.95 | -66.28 | -65.98 | -67.99 | -62.55 | -69.79 |

| BA.2 | 1 | 2 | 3 | 4 | 5 | 6 | 7 | 8 | 9 | 10 |
|---|---|---|---|---|---|---|---|---|---|---|
| $\Delta G_{complex}$ | -11481.57 | -11505.14 | -11533.75 | -11508.50 | -11526.37 | -11540.57 | -11503.02 | -11489.69 | -11471.09 | -11528.86 |
| $\Delta G_{ACE2}$ | -8573.36 | -8580.55 | -8630.48 | -8597.52 | -8626.23 | -8635.78 | -8612.76 | -8590.14 | -8566.48 | -8641.88 |
| $\Delta G_{RBD}$ | -2836.58 | -2855.66 | -2828.29 | -2843.20 | -2832.53 | -2828.14 | -2819.24 | -2829.86 | -2835.02 | -2820.43 |
| $\Delta G_{binding}$ | -71.63 | -68.93 | -74.99 | -67.78 | -67.60 | -76.64 | -71.03 | -69.70 | -69.59 | -66.55 |

| BA.3 | 1 | 2 | 3 | 4 | 5 | 6 | 7 | 8 | 9 | 10 |
|---|---|---|---|---|---|---|---|---|---|---|
| $\Delta G_{complex}$ | -11453.28 | -11488.23 | -11444.55 | -11423.93 | -11490.26 | -11481.64 | -11484.93 | -11493.33 | -11429.96 | -11451.42 |
| $\Delta G_{ACE2}$ | -8572.59 | -8592.52 | -8566.63 | -8537.38 | -8592.66 | -8595.85 | -8595.12 | -8617.16 | -8558.73 | -8588.91 |
| $\Delta G_{RBD}$ | -2810.29 | -2812.06 | -2796.32 | -2802.97 | -2813.05 | -2803.66 | -2822.58 | -2793.57 | -2787.09 | -2784.58 |
| $\Delta G_{binding}$ | -70.40 | -83.66 | -81.59 | -83.58 | -84.56 | -82.13 | -67.22 | -82.61 | -84.14 | -77.94 |

| BA.4/5 | 1 | 2 | 3 | 4 | 5 | 6 | 7 | 8 | 9 | 10 |
|---|---|---|---|---|---|---|---|---|---|---|
| $\Delta G_{complex}$ | -11244.78 | -11266.84 | -11203.28 | -11219.91 | -11262.32 | -11246.31 | -11221.65 | -11265.38 | -11229.36 | -11290.83 |
| $\Delta G_{ACE2}$ | -8575.99 | -8597.03 | -8550.16 | -8563.70 | -8602.27 | -8575.22 | -8568.01 | -8599.21 | -8574.57 | -8622.03 |
| $\Delta G_{RBD}$ | -2613.84 | -2616.50 | -2608.88 | -2605.20 | -2607.27 | -2623.48 | -2596.73 | -2610.44 | -2607.87 | -2615.62 |
| $\Delta G_{binding}$ | -54.96 | -53.30 | -44.24 | -51.02 | -52.78 | -47.61 | -56.91 | -55.73 | -46.93 | -53.18 |

**Note:** $\Delta G_{binding} = \Delta G_{complex} - (\Delta G_{ACE2} + \Delta G_{RBD})$

**Table S2.** Residue contacts between $RBD_{WT}$ and ACE2.

| R1 | I1 | C1 | R2 | I2 | C2 | dCA | OV | rCSU |
|---|---|---|---|---|---|---|---|---|
| SER | 19 | A | ALA | 475 | E | 0.75229 | | rCSU |
| GLN | 24 | A | ALA | 475 | E | 0.6113 | OV | |
| GLN | 24 | A | GLY | 476 | E | 0.6489 | OV | |
| GLN | 24 | A | ASN | 487 | E | 0.70125 | OV | rCSU |
| THR | 27 | A | PHE | 456 | E | 0.84427 | OV | rCSU |
| THR | 27 | A | TYR | 473 | E | 1.02857 | OV | rCSU |
| THR | 27 | A | ALA | 475 | E | 0.7051 | OV | rCSU |
| THR | 27 | A | TYR | 489 | E | 0.92876 | OV | rCSU |
| PHE | 28 | A | TYR | 489 | E | 0.88592 | OV | |
| ASP | 30 | A | LYS | 417 | E | 1.09585 | OV | rCSU |
| ASP | 30 | A | LEU | 455 | E | 0.84261 | OV | |
| ASP | 30 | A | PHE | 456 | E | 0.91076 | OV | rCSU |
| LYS | 31 | A | LEU | 455 | E | 0.80952 | OV | rCSU |
| LYS | 31 | A | PHE | 456 | E | 0.91202 | OV | rCSU |
| LYS | 31 | A | TYR | 489 | E | 0.79262 | OV | rCSU |
| LYS | 31 | A | GLN | 493 | E | 0.88441 | OV | |
| HIS | 34 | A | LEU | 455 | E | 0.91561 | OV | |
| HIS | 34 | A | GLN | 493 | E | 0.7895 | OV | |
| GLU | 35 | A | GLN | 493 | E | 0.8442 | OV | rCSU |
| ASP | 38 | A | TYR | 449 | E | 1.06818 | OV | rCSU |
| ASP | 38 | A | GLY | 496 | E | 0.67724 | OV | |
| TYR | 41 | A | GLN | 498 | E | 0.96852 | OV | |
| TYR | 41 | A | THR | 500 | E | 1.0422 | OV | rCSU |
| TYR | 41 | A | ASN | 501 | E | 0.9389 | OV | rCSU |
| GLN | 42 | A | GLY | 446 | E | 0.76589 | OV | rCSU |
| GLN | 42 | A | GLN | 498 | E | 0.97889 | OV | |
| LEU | 45 | A | VAL | 445 | E | 1.014 | | rCSU |
| LEU | 45 | A | GLN | 498 | E | 1.03427 | OV | rCSU |
| LEU | 79 | A | PHE | 486 | E | 0.74839 | OV | rCSU |
| MET | 82 | A | PHE | 486 | E | 0.90662 | OV | rCSU |
| TYR | 83 | A | PHE | 486 | E | 1.0725 | OV | |
| TYR | 83 | A | ASN | 487 | E | 1.06227 | OV | rCSU |
| ASN | 330 | A | THR | 500 | E | 0.74946 | OV | |
| LYS | 353 | A | GLY | 496 | E | 0.96303 | OV | rCSU |
| LYS | 353 | A | ASN | 501 | E | 0.55088 | OV | |
| LYS | 353 | A | GLY | 502 | E | 0.60388 | OV | rCSU |
| LYS | 353 | A | TYR | 505 | E | 0.57407 | OV | rCSU |
| GLY | 354 | A | GLY | 502 | E | 0.41146 | OV | |
| GLY | 354 | A | TYR | 505 | E | 0.6621 | OV | |
| ASP | 355 | A | THR | 500 | E | 0.66401 | OV | |
| ASP | 355 | A | ASN | 501 | E | 0.52684 | OV | |
| ARG | 357 | A | THR | 500 | E | 1.05413 | OV | |
| GLN | 498 | E | LYS | 353 | A | 0.94004 | | rCSU |
| PRO | 499 | E | ASN | 330 | A | 1.05902 | | rCSU |
| THR | 500 | E | LEU | 45 | A | 0.95691 | | rCSU |

**Note:** dCA is distance between C$\alpha$ atom centers (nm); R, I, C are residue name, residue index from PDB file, chain from PDB file, respectively; OV and rCSU represent overlap and repulsive contacts of structural units, respectively. A and E represent ACE2 and $RBD_{WT}$, respectively.

**Table S3.** Residue contacts between $RBD_{BA.1}$ and ACE2.

| R1 | I1 | C1 | R2 | I2 | C2 | dCA | OV | rCSU |
|---|---|---|---|---|---|---|---|---|
| SER | 19 | A | ALA | 475 | E | 0.73257 | | rCSU |
| SER | 19 | A | ASN | 477 | E | 0.76654 | | rCSU |
| GLN | 24 | A | ASN | 487 | E | 0.68265 | OV | |
| THR | 27 | A | PHE | 456 | E | 0.84041 | OV | rCSU |
| THR | 27 | A | TYR | 473 | E | 1.02576 | OV | rCSU |
| THR | 27 | A | ALA | 475 | E | 0.74016 | OV | |
| THR | 27 | A | TYR | 489 | E | 0.89666 | OV | rCSU |
| PHE | 28 | A | TYR | 489 | E | 0.85576 | OV | |
| ASP | 30 | A | PHE | 456 | E | 0.90331 | OV | |
| LYS | 31 | A | PHE | 456 | E | 0.90805 | OV | rCSU |
| LYS | 31 | A | TYR | 489 | E | 0.77296 | OV | rCSU |
| LYS | 31 | A | ARG | 493 | E | 0.88681 | OV | |
| HIS | 34 | A | LEU | 455 | E | 0.90829 | OV | rCSU |
| HIS | 34 | A | ARG | 493 | E | 0.79079 | OV | rCSU |
| GLU | 35 | A | ARG | 493 | E | 0.84311 | OV | rCSU |
| ASP | 38 | A | TYR | 449 | E | 1.07691 | OV | rCSU |
| ASP | 38 | A | SER | 496 | E | 0.68221 | OV | rCSU |
| ASP | 38 | A | TYR | 501 | E | 1.09515 | | rCSU |
| TYR | 41 | A | ARG | 498 | E | 0.97618 | OV | |
| TYR | 41 | A | THR | 500 | E | 1.03513 | OV | rCSU |
| TYR | 41 | A | TYR | 501 | E | 0.94865 | OV | |
| GLN | 42 | A | SER | 446 | E | 0.76927 | OV | rCSU |
| GLN | 42 | A | ARG | 498 | E | 0.98681 | OV | rCSU |
| LEU | 45 | A | VAL | 445 | E | 1.02889 | | rCSU |
| LEU | 45 | A | ARG | 498 | E | 1.04259 | OV | |
| LEU | 45 | A | THR | 500 | E | 0.95 | | rCSU |
| LEU | 79 | A | PHE | 486 | E | 0.76326 | OV | rCSU |
| MET | 82 | A | PHE | 486 | E | 0.8984 | OV | rCSU |
| TYR | 83 | A | PHE | 486 | E | 1.05541 | OV | rCSU |
| TYR | 83 | A | ASN | 487 | E | 1.06018 | OV | rCSU |
| ASN | 330 | A | THR | 500 | E | 0.75623 | OV | rCSU |
| LYS | 353 | A | SER | 496 | E | 0.96394 | OV | rCSU |
| LYS | 353 | A | TYR | 501 | E | 0.55955 | OV | rCSU |
| LYS | 353 | A | GLY | 502 | E | 0.6196 | OV | rCSU |
| LYS | 353 | A | HIS | 505 | E | 0.56904 | OV | |
| GLY | 354 | A | GLY | 502 | E | 0.42931 | OV | |
| GLY | 354 | A | HIS | 505 | E | 0.65534 | OV | |
| ASP | 355 | A | THR | 500 | E | 0.6687 | OV | |
| ARG | 357 | A | THR | 500 | E | 1.0601 | OV | |
| ALA | 475 | E | GLN | 24 | A | 0.62967 | | rCSU |
| PRO | 499 | E | ASN | 330 | A | 1.05954 | | rCSU |

**Note:** dCA is distance between C$\alpha$ atom centers (nm); R, I, C are residue name, residue index from PDB file, chain from PDB file, respectively; OV and rCSU represent overlap and repulsive contacts of structural units, respectively. A and E represent ACE2 and $RBD_{BA.1}$, respectively.

**Table S4.** Residue contacts between $RBD_{BA.2}$ and ACE2.

| R1 | I1 | C1 | R2 | I2 | C2 | dCA | OV | rCSU |
|---|---|---|---|---|---|---|---|---|
| SER | 19 | A | ALA | 475 | E | 0.7414 | | rCSU |
| SER | 19 | A | ASN | 477 | E | 0.74397 | | rCSU |
| THR | 27 | A | PHE | 456 | E | 0.84915 | OV | rCSU |
| THR | 27 | A | ALA | 475 | E | 0.7318 | OV | rCSU |
| THR | 27 | A | TYR | 489 | E | 0.9071 | OV | rCSU |
| PHE | 28 | A | TYR | 489 | E | 0.87132 | OV | |
| ASP | 30 | A | LEU | 455 | E | 0.84207 | OV | |
| ASP | 30 | A | PHE | 456 | E | 0.91235 | OV | |
| LYS | 31 | A | LEU | 455 | E | 0.80869 | OV | rCSU |
| LYS | 31 | A | PHE | 456 | E | 0.91669 | | rCSU |
| LYS | 31 | A | TYR | 489 | E | 0.7804 | OV | rCSU |
| LYS | 31 | A | PHE | 490 | E | 0.94887 | | rCSU |
| LYS | 31 | A | ARG | 493 | E | 0.88059 | OV | |
| HIS | 34 | A | LEU | 455 | E | 0.91361 | OV | |
| HIS | 34 | A | ARG | 493 | E | 0.78516 | OV | |
| GLU | 35 | A | ARG | 493 | E | 0.84314 | OV | rCSU |
| ASP | 38 | A | TYR | 449 | E | 1.07476 | OV | rCSU |
| ASP | 38 | A | SER | 496 | E | 0.67834 | OV | rCSU |
| ASP | 38 | A | ARG | 498 | E | 1.02157 | | rCSU |
| TYR | 41 | A | ARG | 498 | E | 0.98166 | OV | |
| TYR | 41 | A | THR | 500 | E | 1.01994 | OV | rCSU |
| TYR | 41 | A | TYR | 501 | E | 0.94538 | OV | |
| GLN | 42 | A | SER | 446 | E | 0.77469 | OV | rCSU |
| GLN | 42 | A | ARG | 498 | E | 0.99069 | OV | rCSU |
| LEU | 45 | A | VAL | 445 | E | 1.02372 | | rCSU |
| LEU | 45 | A | ARG | 498 | E | 1.04659 | OV | |
| LEU | 45 | A | THR | 500 | E | 0.93795 | | rCSU |
| LEU | 79 | A | PHE | 486 | E | 0.75704 | OV | rCSU |
| MET | 82 | A | PHE | 486 | E | 0.91149 | OV | rCSU |
| TYR | 83 | A | PHE | 486 | E | 1.06632 | OV | |
| ASN | 330 | A | THR | 500 | E | 0.75875 | OV | rCSU |
| LYS | 353 | A | SER | 496 | E | 0.96094 | OV | rCSU |
| LYS | 353 | A | TYR | 501 | E | 0.55081 | OV | |
| LYS | 353 | A | GLY | 502 | E | 0.62249 | OV | rCSU |
| LYS | 353 | A | HIS | 505 | E | 0.56394 | OV | |
| GLY | 354 | A | GLY | 502 | E | 0.42915 | OV | |
| GLY | 354 | A | HIS | 505 | E | 0.64695 | OV | rCSU |
| ASP | 355 | A | THR | 500 | E | 0.65862 | OV | |
| ARG | 357 | A | THR | 500 | E | 1.05404 | OV | |
| TYR | 473 | E | THR | 27 | A | 1.04115 | | rCSU |
| ALA | 475 | E | GLN | 24 | A | 0.63172 | | rCSU |
| PRO | 499 | E | ASN | 330 | A | 1.0525 | | rCSU |

**Note:** dCA is distance between C$\alpha$ atom centers (nm); R, I, C are residue name, residue index from PDB file, chain from PDB file, respectively; OV and rCSU represent overlap and repulsive contacts of structural units, respectively. A and E represent ACE2 and $RBD_{BA.2}$, respectively.

**Table S5.** Residue contacts between $RBD_{BA.3}$ and ACE2.

| R1 | I1 | C1 | R2 | I2 | C2 | dCA | OV | rCSU |
|---|---|---|---|---|---|---|---|---|
| SER | 19 | A | ALA | 475 | E | 0.76564 | | rCSU |
| GLN | 24 | A | ALA | 475 | E | 0.58638 | OV | |
| GLN | 24 | A | GLY | 476 | E | 0.6043 | OV | |
| GLN | 24 | A | ASN | 487 | E | 0.71378 | OV | rCSU |
| THR | 27 | A | PHE | 456 | E | 0.85135 | OV | rCSU |
| THR | 27 | A | TYR | 473 | E | 1.01428 | OV | rCSU |
| THR | 27 | A | ALA | 475 | E | 0.66749 | OV | rCSU |
| THR | 27 | A | TYR | 489 | E | 0.93772 | | rCSU |
| PHE | 28 | A | TYR | 489 | E | 0.89201 | OV | |
| ASP | 30 | A | PHE | 456 | E | 0.91062 | OV | rCSU |
| LYS | 31 | A | LEU | 455 | E | 0.80797 | OV | rCSU |
| LYS | 31 | A | PHE | 456 | E | 0.91148 | OV | rCSU |
| LYS | 31 | A | TYR | 489 | E | 0.7919 | OV | rCSU |
| LYS | 31 | A | PHE | 490 | E | 0.9732 | | rCSU |
| LYS | 31 | A | ARG | 493 | E | 0.8968 | OV | rCSU |
| HIS | 34 | A | LEU | 455 | E | 0.91069 | OV | |
| HIS | 34 | A | ARG | 493 | E | 0.8047 | OV | |
| GLU | 35 | A | ARG | 493 | E | 0.8558 | OV | rCSU |
| ASP | 38 | A | TYR | 449 | E | 1.07603 | OV | rCSU |
| ASP | 38 | A | TYR | 501 | E | 1.0922 | OV | rCSU |
| TYR | 41 | A | ARG | 498 | E | 0.95902 | OV | |
| TYR | 41 | A | THR | 500 | E | 1.01586 | OV | |
| TYR | 41 | A | TYR | 501 | E | 0.94683 | OV | |
| GLN | 42 | A | SER | 446 | E | 0.77972 | OV | rCSU |
| LEU | 45 | A | VAL | 445 | E | 1.04234 | | rCSU |
| LEU | 45 | A | ARG | 498 | E | 1.03044 | OV | |
| LEU | 79 | A | PHE | 486 | E | 0.73951 | OV | rCSU |
| MET | 82 | A | PHE | 486 | E | 0.92858 | OV | rCSU |
| TYR | 83 | A | PHE | 486 | E | 1.09703 | OV | rCSU |
| TYR | 83 | A | ASN | 487 | E | 1.0637 | OV | rCSU |
| ASN | 330 | A | THR | 500 | E | 0.75914 | OV | |
| LYS | 353 | A | GLY | 496 | E | 0.96315 | OV | rCSU |
| LYS | 353 | A | TYR | 501 | E | 0.5507 | OV | rCSU |
| LYS | 353 | A | GLY | 502 | E | 0.61642 | OV | rCSU |
| LYS | 353 | A | HIS | 505 | E | 0.56038 | OV | rCSU |
| GLY | 354 | A | GLY | 502 | E | 0.42252 | OV | |
| GLY | 354 | A | HIS | 505 | E | 0.64782 | OV | |
| ASP | 355 | A | ARG | 498 | E | 1.01039 | OV | rCSU |
| ASP | 355 | A | THR | 500 | E | 0.65501 | OV | |
| ASP | 355 | A | HIS | 505 | E | 0.88167 | OV | |
| ARG | 357 | A | THR | 500 | E | 1.05173 | OV | |
| PHE | 486 | E | PHE | 28 | A | 0.88106 | | rCSU |
| PRO | 499 | E | ASN | 330 | A | 1.05348 | | rCSU |

**Note:** dCA is distance between C$\alpha$ atom centers (nm); R, I, C are residue name, residue index from PDB file, chain from PDB file, respectively; OV and rCSU represent overlap and repulsive contacts of structural units, respectively. A and E represent ACE2 and $RBD_{BA.3}$, respectively.

**Table S6.** Residue contacts between $RBD_{BA.4/5}$ and ACE2.

| R1 | I1 | C1 | R2 | I2 | C2 | dCA | OV | rCSU |
|---|---|---|---|---|---|---|---|---|
| SER | 19 | A | ALA | 475 | E | 0.76123 | | rCSU |
| SER | 19 | A | ASN | 477 | E | 0.69884 | | rCSU |
| GLN | 24 | A | GLY | 476 | E | 0.65779 | OV | |
| GLN | 24 | A | ASN | 487 | E | 0.71914 | OV | rCSU |
| THR | 27 | A | PHE | 456 | E | 0.84928 | OV | rCSU |
| THR | 27 | A | ALA | 475 | E | 0.70444 | OV | rCSU |
| THR | 27 | A | TYR | 489 | E | 0.93798 | OV | rCSU |
| PHE | 28 | A | TYR | 489 | E | 0.89614 | OV | |
| ASP | 30 | A | LEU | 455 | E | 0.84233 | OV | |
| ASP | 30 | A | PHE | 456 | E | 0.90955 | OV | |
| LYS | 31 | A | LEU | 455 | E | 0.80545 | OV | |
| LYS | 31 | A | PHE | 456 | E | 0.91015 | OV | rCSU |
| LYS | 31 | A | TYR | 489 | E | 0.80112 | OV | rCSU |
| LYS | 31 | A | GLN | 493 | E | 0.89182 | OV | rCSU |
| HIS | 34 | A | LEU | 455 | E | 0.9087 | OV | |
| HIS | 34 | A | GLN | 493 | E | 0.79727 | | rCSU |
| ASP | 38 | A | TYR | 449 | E | 1.07957 | | rCSU |
| TYR | 41 | A | ARG | 498 | E | 0.97133 | OV | |
| TYR | 41 | A | THR | 500 | E | 1.03272 | OV | rCSU |
| TYR | 41 | A | TYR | 501 | E | 0.9527 | OV | |
| GLN | 42 | A | GLY | 446 | E | 0.80096 | OV | rCSU |
| GLN | 42 | A | ARG | 498 | E | 0.98389 | | rCSU |
| LEU | 45 | A | ARG | 498 | E | 1.03826 | OV | |
| LEU | 45 | A | THR | 500 | E | 0.94675 | | rCSU |
| LEU | 79 | A | VAL | 486 | E | 0.77634 | OV | rCSU |
| MET | 82 | A | VAL | 486 | E | 0.94175 | OV | rCSU |
| TYR | 83 | A | ASN | 487 | E | 1.0827 | OV | rCSU |
| ASN | 330 | A | THR | 500 | E | 0.75205 | OV | rCSU |
| GLY | 352 | A | HIS | 505 | E | 0.94037 | OV | |
| LYS | 353 | A | GLY | 496 | E | 0.96183 | OV | rCSU |
| LYS | 353 | A | TYR | 501 | E | 0.56043 | OV | rCSU |
| LYS | 353 | A | GLY | 502 | E | 0.6228 | OV | rCSU |
| LYS | 353 | A | HIS | 505 | E | 0.56175 | OV | rCSU |
| GLY | 354 | A | GLY | 502 | E | 0.42766 | OV | |
| GLY | 354 | A | HIS | 505 | E | 0.64553 | OV | |
| ASP | 355 | A | THR | 500 | E | 0.66401 | OV | |
| ARG | 357 | A | THR | 500 | E | 1.05436 | OV | |
| TYR | 473 | E | THR | 27 | A | 1.03366 | | rCSU |
| PRO | 499 | E | ASN | 330 | A | 1.05474 | | rCSU |

**Note:** dCA is distance between C$\alpha$ atom centers (nm); R, I, C are residue name, residue index from PDB file, chain from PDB file, respectively; OV and rCSU represent overlap and repulsive contacts of structural units, respectively. A and E represent ACE2 and $RBD_{BA.4/5}$, respectively.

**Table S7.** Several quantity information in MD simulation setup.

| RBD-ACE2 | NNC[a] | NIN[b] | | NWM[c] | EMS[d] |
|---|---|---|---|---|---|
| | | NA | CL | | |
| WT | -25 | 191 | 166 | 56056 | 1388 |
| BA.1 | -22 | 188 | 166 | 55983 | 1245 |
| BA.2 | -22 | 190 | 168 | 56472 | 1403 |
| BA.3 | -20 | 186 | 166 | 55985 | 1460 |
| BA.4/5 | -22 | 187 | 165 | 55649 | 1293 |

[a]Number of net charges.

[b]Number of ions.

[c]Number of water molecules.

[d]Number of energy minimization steps

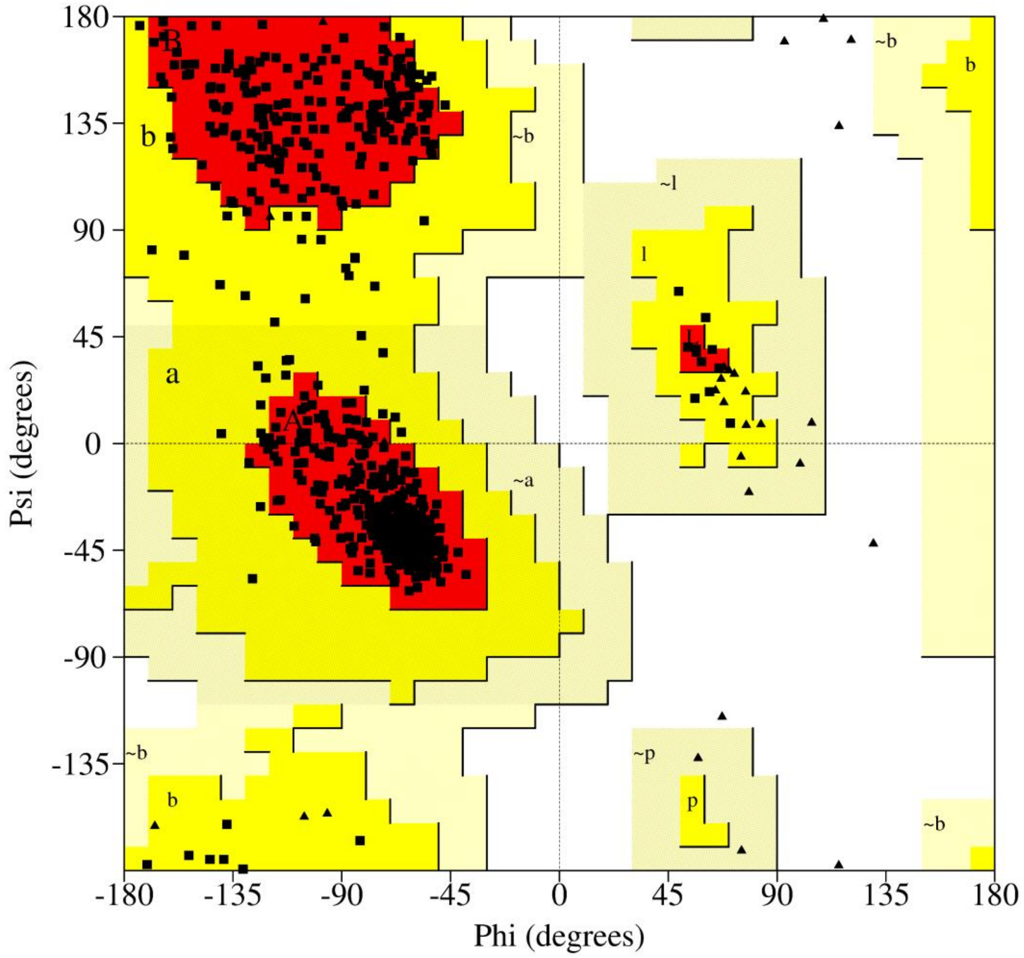


Plot statistics

| | | |
|---|---|---|
| Residues in most favoured regions [A,B,L] | 650 | 92.3% |
| Residues in additional allowed regions [a,b,l,p] | 54 | 7.7% |
| Residues in generously allowed regions [~a,~b,~l,~p] | 0 | 0.0% |
| Residues in disallowed regions | 0 | 0.0% |
| | ---- | ------ |
| Number of non-glycine and non-proline residues | 704 | 100.0% |
| Number of end-residues (excl. Gly and Pro) | 3 | |
| Number of glycine residues (shown as triangles) | 47 | |
| Number of proline residues | 37 | |
| | ---- | |
| Total number of residues | 791 | |

Based on an analysis of 118 structures of resolution of at least 2.0 Angstroms and R-factor no greater than 20%, a good quality model would be expected to have over 90% in the most favoured regions.

**Figure S3.** Ramachandran plot of $RBD_{WT}$-ACE2 complex structure

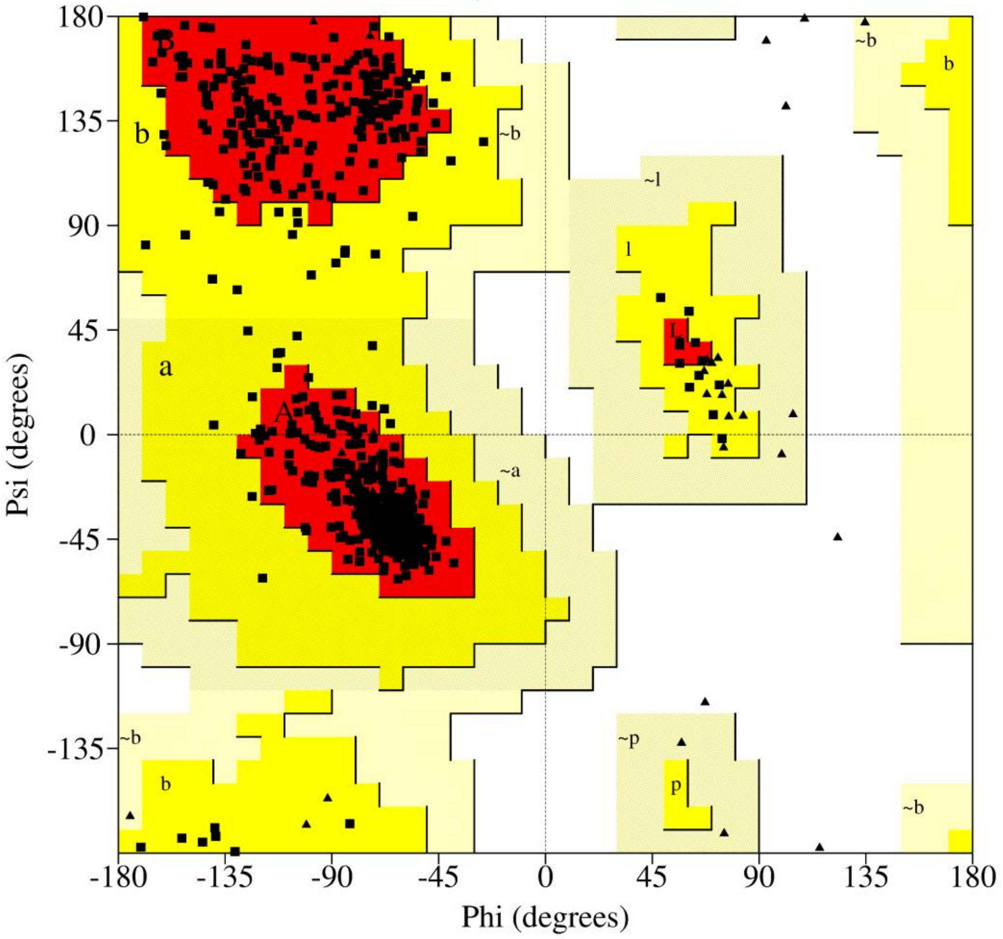


Plot statistics

| | | |
|---|---|---|
| Residues in most favoured regions [A,B,L] | 652 | 92.4% |
| Residues in additional allowed regions [a,b,l,p] | 54 | 7.6% |
| Residues in generously allowed regions [~a,~b,~l,~p] | 0 | 0.0% |
| Residues in disallowed regions | 0 | 0.0% |
| | ---- | ------ |
| Number of non-glycine and non-proline residues | 706 | 100.0% |
| Number of end-residues (excl. Gly and Pro) | 3 | |
| Number of glycine residues (shown as triangles) | 44 | |
| Number of proline residues | 38 | |
| | ---- | |
| Total number of residues | 791 | |

Based on an analysis of 118 structures of resolution of at least 2.0 Angstroms and R-factor no greater than 20%, a good quality model would be expected to have over 90% in the most favoured regions.

**Figure S4.** Ramachandran plot of $RBD_{BA.1}$-ACE2 complex structure

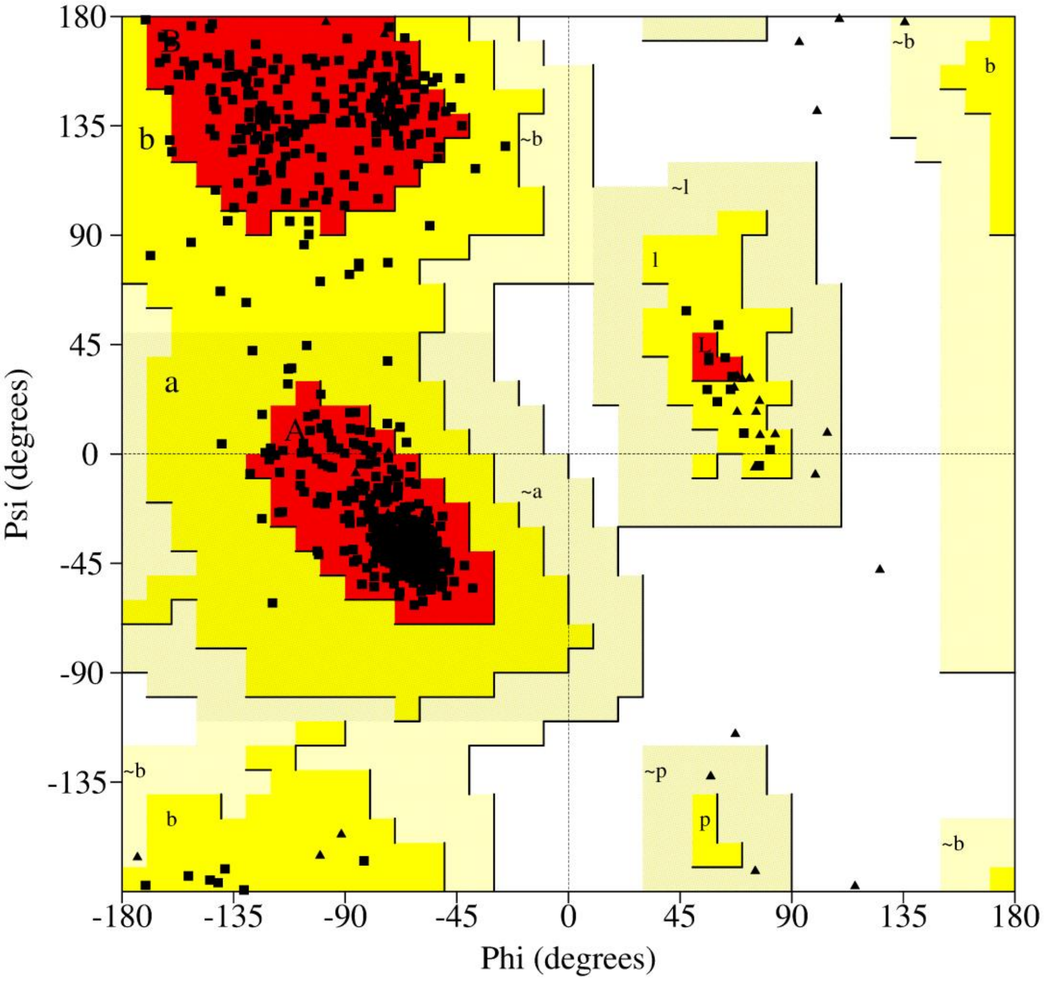


Plot statistics

| | | |
|---|---|---|
| Residues in most favoured regions [A,B,L] | 649 | 91.9% |
| Residues in additional allowed regions [a,b,l,p] | 57 | 8.1% |
| Residues in generously allowed regions [~a,~b,~l,~p] | 0 | 0.0% |
| Residues in disallowed regions | 0 | 0.0% |
| | ---- | ------ |
| Number of non-glycine and non-proline residues | 706 | 100.0% |
| Number of end-residues (excl. Gly and Pro) | 3 | |
| Number of glycine residues (shown as triangles) | 44 | |
| Number of proline residues | 38 | |
| | ---- | |
| Total number of residues | 791 | |

Based on an analysis of 118 structures of resolution of at least 2.0 Angstroms and R-factor no greater than 20%, a good quality model would be expected to have over 90% in the most favoured regions.

**Figure S5.** Ramachandran plot of $RBD_{BA.2}$-ACE2 complex structure

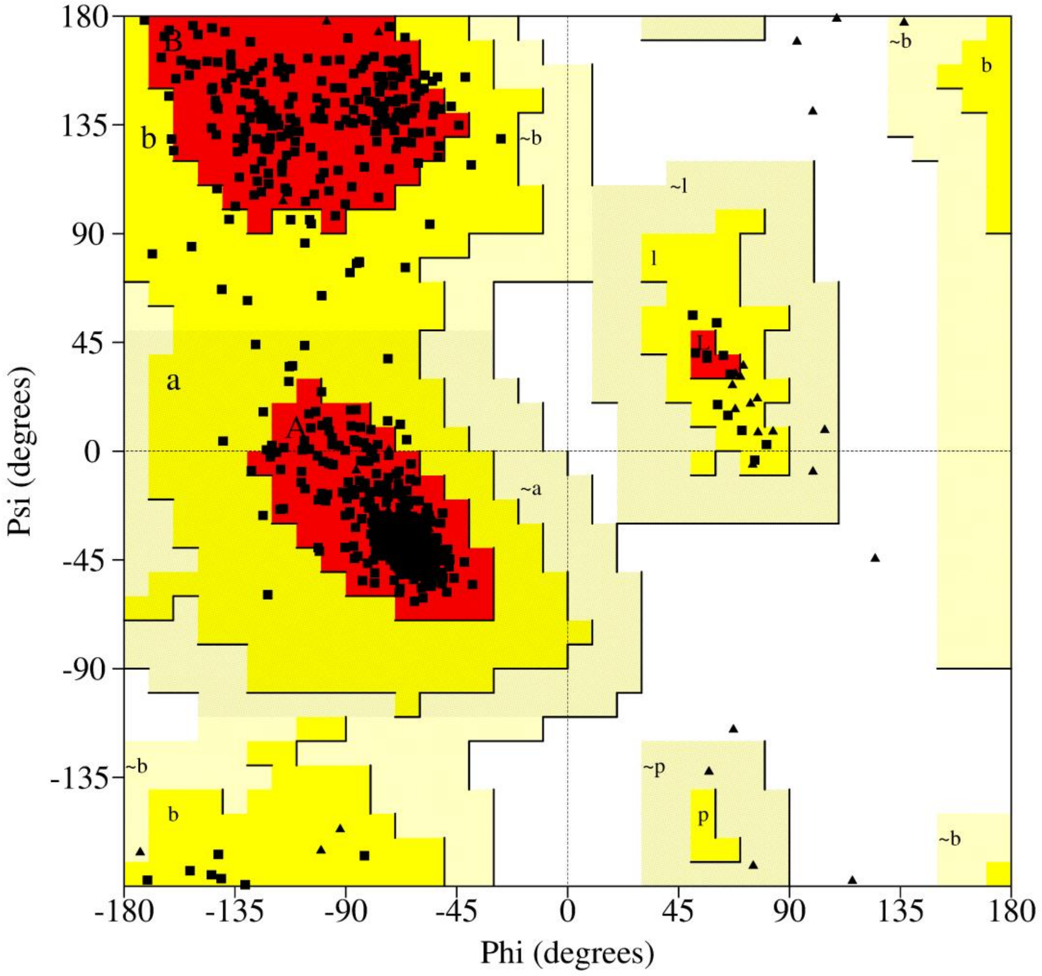


Plot statistics

| | | |
|---|---|---|
| Residues in most favoured regions [A,B,L] | 650 | 92.2% |
| Residues in additional allowed regions [a,b,l,p] | 55 | 7.8% |
| Residues in generously allowed regions [~a,~b,~l,~p] | 0 | 0.0% |
| Residues in disallowed regions | 0 | 0.0% |
| | ---- | ------ |
| Number of non-glycine and non-proline residues | 705 | 100.0% |
| Number of end-residues (excl. Gly and Pro) | 3 | |
| Number of glycine residues (shown as triangles) | 45 | |
| Number of proline residues | 38 | |
| | ---- | |
| Total number of residues | 791 | |

Based on an analysis of 118 structures of resolution of at least 2.0 Angstroms and R-factor no greater than 20%, a good quality model would be expected to have over 90% in the most favoured regions.

**Figure S6.** Ramachandran plot of RBD$_{BA.3}$-ACE2 complex structure

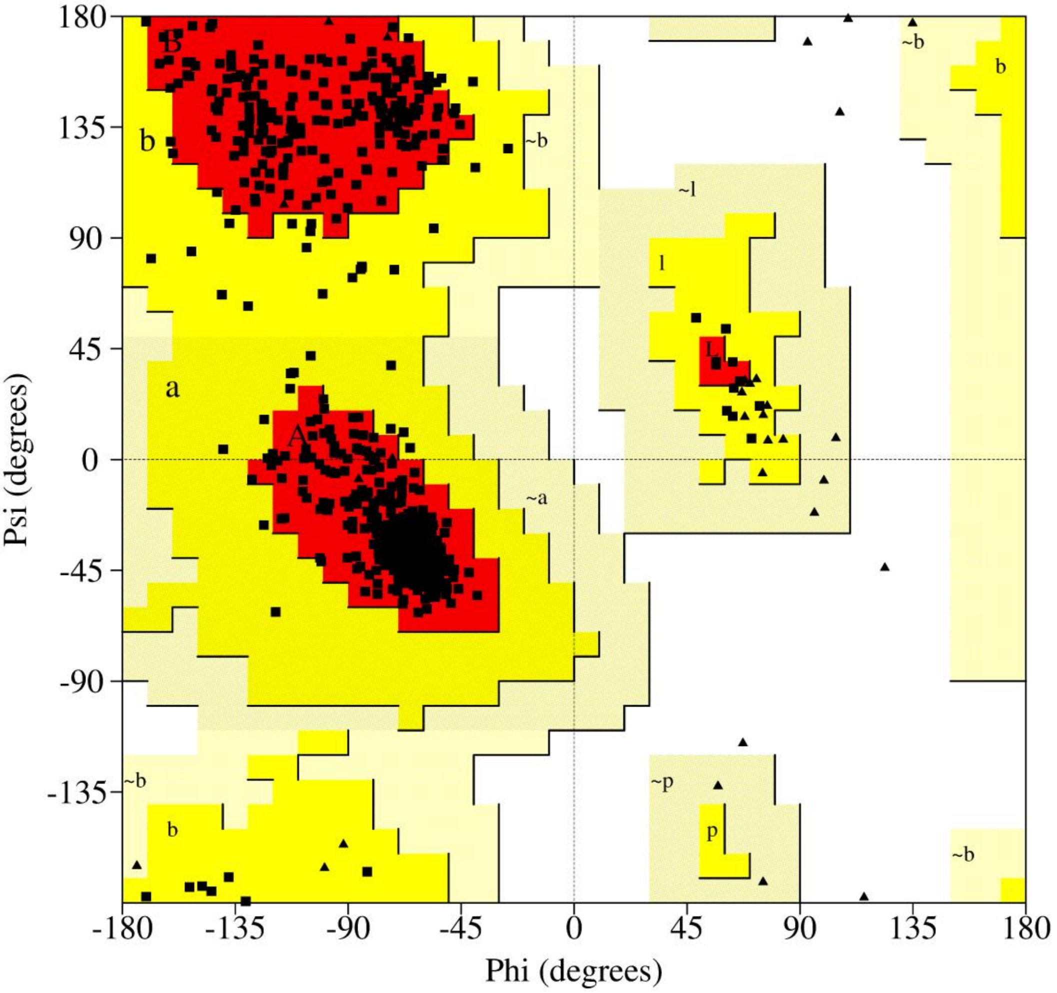


Plot statistics

| | | |
|---|---|---|
| Residues in most favoured regions [A,B,L] | 649 | 92.2% |
| Residues in additional allowed regions [a,b,l,p] | 55 | 7.8% |
| Residues in generously allowed regions [~a,~b,~l,~p] | 0 | 0.0% |
| Residues in disallowed regions | 0 | 0.0% |
| | ---- | ------ |
| Number of non-glycine and non-proline residues | 704 | 100.0% |
| Number of end-residues (excl. Gly and Pro) | 3 | |
| Number of glycine residues (shown as triangles) | 46 | |
| Number of proline residues | 38 | |
| | ---- | |
| Total number of residues | 791 | |

Based on an analysis of 118 structures of resolution of at least 2.0 Angstroms and R-factor no greater than 20%, a good quality model would be expected to have over 90% in the most favoured regions.

**Figure S7.** Ramachandran plot of $RBD_{BA.4/5}$-ACE2 complex structure
